# Time-Course RNAseq Reveals *Exserohilum turcicum* Effectors and Pathogenicity Determinants

**DOI:** 10.3389/fmicb.2020.00360

**Published:** 2020-03-20

**Authors:** Maria Petronella Human, Dave Kenneth Berger, Bridget Genevieve Crampton

**Affiliations:** Department of Plant and Soil Sciences, Forestry and Agricultural Biotechnology Institute (FABI), University of Pretoria, Pretoria, South Africa

**Keywords:** SIX13-like, AVRHt1, Ecp6, transcriptional profiling, effector sequencing

## Abstract

*Exserohilum turcicum* (sexual stage *Setosphaeria turcica*) is the hemibiotrophic causal agent of northern leaf blight of maize and sorghum. This study aimed to identify the genes involved in host colonization during the biotrophic and necrotrophic phases of infection. It also aimed to identify race-specific differences in gene expression. RNAseq of maize seedlings inoculated with a race 13N or 23N *E. turcicum* isolate was conducted before inoculation and at 2, 5, 7, and 13 days post-inoculation (dpi). Biological replicates were pooled per time point for each race and sequenced. A bioinformatics pipeline was used to identify candidate effectors, and expression was validated for selected candidates. Fungal biomass was positively correlated with the percentages of *E. turcicum* reads mapped, which were low at early time points (2–7 dpi) with a significant increase at 13 dpi, indicating a lifestyle switch from biotrophy to necrotrophy between 7 and 13 dpi. *AVRHt1* is the putative *E. turcicum* effector recognized by the maize resistance gene *Ht1*. Consistent with this, *AVRHt1* was expressed *in planta* by race 23N, but transcripts were absent in race 13N. In addition, specific transposable elements were expressed in 23N only. Genes encoding the virulence-associated peptidases leupeptin-inhibiting protein 1 and fungalysin were expressed *in planta*. Transcriptional profiles of genes involved in secondary metabolite synthesis or cell wall degradation revealed the importance of these genes during late stages of infection (13 dpi). A total of 346 expressed candidate effectors were identified, including Ecp6 and proteins similar to the secreted in xylem (SIX) effectors common to *formae speciales* of *Fusarium oxysporum*, SIX13 and SIX5. Expression profiling of *Ecp6* and *SIX13-like* indicated a peak in expression at 5 and 7 dpi compared to 2 and 13 dpi. Sequencing of *SIX13-like* from diverse isolates of *E. turcicum* revealed host-specific polymorphisms that were mostly non-synonymous, resulting in two groups of SIX13-like proteins that corresponded to the maize or sorghum origin of each isolate. This study suggests putative mechanisms whereby *E. turcicum* causes disease. Identification of the candidate effector *SIX13-like* is consistent with the infection mode of *E. turcicum* through the xylem of susceptible hosts.

## Introduction

*Exserohilum turcicum* (sexual stage *Setosphaeria turcica*) is the causal agent of northern leaf blight (NLB), a destructive foliar disease of maize, sorghum, and related grass species (Agrios, 2005). In maize, four major resistance (*R-*) genes have been characterized, which convey resistance to *E. turcicum*, namely, *Ht1, Ht2, Ht3*, and *HtN* (Galiano-Carneiro and Miedaner, 2017)*. E. turcicum* races are classified based on their ability to overcome these *R*-genes, e.g., a race 13N isolate can overcome the resistance of maize plants provided by the *Ht1, Ht3*, and *HtN R-*genes (Leonard et al., 1989). Similarly, maize plants carrying the *Ht2, Ht3*, and *HtN R-*genes are susceptible to race 23N *E. turcicum* isolates.

Host specificity of *E. turcicum* has been observed in isolates from maize, sorghum, and Johnson grass (Bhowmik and Prasada, 1970; Bergquist and Masias, 1974). Subsequently, the use of *formae speciales* was suggested for isolates specific to maize (f. sp. *zeae*) or sorghum (f. sp. *sorghi*) (Bergquist and Masias, 1974; Masias and Bergquis, 1974). Inoculation of *E. turcicum* onto maize, sorghum, and Johnson grass showed that the majority of isolates was specific to the host from which the isolates were sampled. However, isolates pathogenic to two or more hosts were also observed, and a third specialization group, f. sp. *complexa*, was suggested to refer to isolates pathogenic to more than one host (Hamid and Aragaki, 1975). Molecular studies comparing random amplified polymorphic DNA (RAPD) profiles of *E. turcicum* isolates from different hosts indicated unique profiles in sorghum isolates not observed in those from maize (Borchardt et al., 1998; Ferguson and Carson, 2004). Recently, Nieuwoudt et al. (2018) applied microsatellite markers to *E. turcicum* isolates from maize and sorghum and demonstrated that these populations are genetically distinct.

The infection strategy of the NLB pathogen was investigated by inoculating susceptible or resistant maize seedlings with *E. turcicum* conidial suspensions (Jennings and Ullstrup, 1957; Hilu and Hooker, 1964, 1965). Germinating conidia produce appressoria, which give rise to penetration pegs that penetrate the maize epidermal cell layers directly (Hilu and Hooker, 1964; Knox-Davies, 1974). After penetration, *E. turcicum* grows in or between epidermal cells and slowly advances in or between adjacent mesophyll cells with some hyphae growing toward the xylem vessels. The hyphae of *E. turcicum* colonized xylem vessels, and at this stage of infection, disease symptoms are limited to light, chlorotic flecks (Hilu and Hooker, 1964; Kotze et al., 2019). In susceptible interactions, fungal proliferation in the xylem is rapid, where after hyphae grow from the xylem into adjacent mesophyll cells, leading to widespread tissue necrosis and characteristic elongated NLB lesions (Jennings and Ullstrup, 1957; Hilu and Hooker, 1964; Kotze et al., 2019). In mono- or polygenic-resistant maize plants, proliferation of *E. turcicum* hyphae in xylem is limited, and small necrotic flecks result from slowly advancing hyphae in the mesophyll (Hilu and Hooker, 1965). Necrosis in maize results from the production of monocerin (a non-specific toxin) by *E. turcicum* to cause plant cell death (Cuq et al., 1993). However, the timing of secretion of this toxin during the infection process remains to be elucidated.

Although *E. turcicum* has been reported as a hemibiotroph (Xue et al., 2013; Hurni et al., 2015), experimental evidence to support this claim is lacking. The difference between biotrophs and necrotrophs is largely ascribed to whether the pathogen uses its own or host resources during early infection stages (Laluk and Mengiste, 2010). Rudd et al. (2015) differentiated these lifestyle strategies in *Zymoseptoria tritici* by assessing upregulation of enzymes in the β-oxidation pathway (Rudd et al., 2015). Upregulation of these genes implies that the pathogen is using internal resources and not following a biotrophic phase of infection.

Hemibiotrophic pathogens stealthily colonize the host during the biotrophic phase by suppressing or evading host defenses to maintain host viability and acquire nutrients (Talbot, 2010; Ohm et al., 2012; Hurni et al., 2015). A necrotrophic stage follows, and host cells are killed due to secretion of toxins and/or degradation enzymes (Oliver and Ipcho, 2004). The exact mechanisms whereby biotrophs obtain nutrients is still unclear; however, evidence suggests that fungi secrete a diverse array of extracellular enzymes that break down host substrates such as lignin, proteins, and lipids into monomeric forms of simple sugars, amino acids, and fatty acids for uptake by the invading pathogen (Talbot, 2010; Meinhardt et al., 2014). Of the fungal genes required for host penetration and colonization, effectors are widely recognized to be instrumental in determining the outcome of host–pathogen interactions (Stergiopoulos and de Wit, 2009; Lo Presti et al., 2015). During biotrophic growth, pathogens secrete effectors that suppress or modulate host defense responses to evade host detection (Lo Presti et al., 2015; Selin et al., 2016). Effectors can also elicit an immune response if the corresponding *R-*gene in the host recognizes and interacts with the corresponding effector (Jones and Dangl, 2006).

Necrotrophy is characterized by increased fungal biomass as well as expression of plant cell wall degrading enzymes (CWDEs) and secondary metabolites to induce host cell death (Laluk and Mengiste, 2010; Palma-Guerrero et al., 2017). Peptidases secreted by fungal pathogens degrade host proteins to provide the invading fungus with cellular energy as well as substrates for growth, cell wall remodeling, protein synthesis, and nucleic acids (Lowe et al., 2015). Plant pathogens secrete CWDEs to aid in host penetration and to invasively colonize the host (Choi et al., 2013). Genes encoding enzymes for secondary metabolite production include polyketide synthases (PKS), non-ribosomal peptide synthases (NPS), terpene synthases (TPS), and hybrid PKS:NPSs (Pusztahelyi et al., 2015). A large number of toxins have been identified from *Dothideomycete* fungi, and in some cases, the PKS or NPS involved in the biosynthesis of these secondary metabolites are known, such as the HC-toxin. Known genes involved in the biosynthesis of HC-toxin in *Cochliobolus carbonum* occur in the *TOX2* locus and include the NPS HC-toxin synthase 1 *(HTS1)* as well as *TOXA, TOXC, TOXD, TOXE, TOXF*, and *TOXG* (Walton, 2006). Although the HC-toxin was considered to be unique to *C. carbonum*, genomic investigations have revealed the presence of *HTS1* in *E. turcicum, Pyrenophora tritici-repentis*, and *Alternaria jesenkae*, of which only *A. jesenkae* has been shown to produce the HC-toxin (Ohm et al., 2012; Manning et al., 2013; Wight et al., 2013).

Sequencing of the *E. turcicum* genome enabled functional annotations and characterization of the peptidase, carbohydrate active enzyme (CAZyme), and secondary metabolite coding ability of this pathogen (Ohm et al., 2012; Condon et al., 2013). However, transcriptome studies of the *E. turcicum*–maize interaction to unravel specific and general gene expression patterns during host invasion are lacking. Furthermore, little investigation of the *E. turcicum* effector repertoire has been undertaken. The conserved effector, *Ecp6*, has been identified and its role in pathogenicity confirmed using a knockout strategy, but expression profiling has not yet been reported (Xue et al., 2013). The *E. turcicum* effector, which is thought to be recognized by the *Ht1* maize *R-*gene, has putatively been identified as a hybrid PKS:NPS termed *AVRHt1* (Mideros et al., 2018). The interaction between *AVRHt1* and *Ht1* gene products is hypothesized to elicit a maize defense response in a similar way to the effector—*R-*gene model (Mideros et al., 2018). A single-nucleotide polymorphism (SNP) was identified in the *AVRHt1* allele present in race 1 isolates, which leads to a premature stop codon. Therefore, the functional protein is not made, and isolates carrying this allele are able to evade host recognition. This SNP is absent in the *AVRHt1* race 2 alleles, which leads to the protein being produced. The *AVRHt1* gene product subsequently interacts with the maize *Ht1 R*-gene product, leading to host resistance. Candidates interacting with the other major maize *R*-genes (*Ht2, Ht3*, and *HtN*) are unknown (Mideros et al., 2018).

In this study, we sequenced and compared the transcriptomes of a race 13N and a race 23N *E. turcicum* isolate during different stages of infection. RNAseq of an *in vitro* grown race 13N isolate was also undertaken. The aim of this study was to identify genes involved in pathogenesis and race specificity, as well as to identify the *E. turcicum* effector repertoire. We hypothesized that effectors will be expressed during biotrophy to establish a compatible interaction with the host, in contrast to the later necrotrophic phase when genes involved in protein degradation, cell wall degradation, and secondary metabolite biosynthesis will be induced. In addition, we hypothesized that expression patterns of pathogenicity-related genes form the basis for race differentiation of this pathogen.

## Materials and Methods

### Maize Inoculation Trial

Conidiating cultures of *E. turcicum* previously characterized as race 13N and 23N were supplied by Dr. M. Craven from the Agricultural Research Council—Grain Crops (Potchefstroom, South Africa) as described previously (Craven and Fourie, 2011). Isolates 2 (race 23N) and 103 (race 13N) were collected from the Free State province of South Africa. Maize Va26 seedlings lacking any *Ht* genes (Leath and Pedersen, 1985) were whorl inoculated at the trifoliar leaf stage with 400 μl of a 9,000 conidia/ml conidial suspension of *E. turcicum* race 23N and 13N on separate plants. Maize leaves were also painted (using a paint brush) with the conidial suspension of either race to ensure that the maize–fungus interaction would be detected in distal parts of the leaves at early time points. Inoculated seedlings were placed into a dew chamber for 16 h where after the seedlings were transferred to a growth chamber. Conditions in the growth chamber were kept at 22°C day and 18°C night temperatures (±2°C) with a flux of 25–50 (362–650 μE m^−2^ s^−1^).

**Figure 1:**
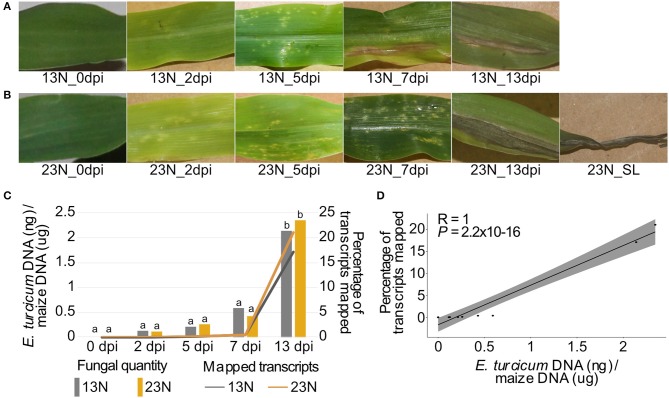
Disease progression, alignment rates, and *in planta* quantification of two *Exserohilum turcicum* races at distinct disease stages. Disease symptoms ranged from light chlorotic flecks to necrotic lesions on maize Va26 seedlings inoculated with a race 13N **(A)** and race 23N **(B)** *E. turcicum* isolates. The rate at which transcripts aligned to the *E. turcicum* Et28A genome was positively correlated with the log-transformed fungal quantities **(C,D)**. The percentages of fungal transcripts mapped is shown on the primary axis, and the log-transformed fungal quantities on the secondary axis. A Tukey multiple pairwise comparison was conducted from a one-way ANOVA to identify significant differences in fungal load between datasets (*P* < 0.05). Different lowercase letters indicate significant differences between datasets.

Samples were harvested by cutting the stem of the seedlings 1 cm above the ground, removing the flag leaf and flash freezing the rest of the plant. Five plants per biological replicate and three biological replicates per time point were collected. Plants were harvested before inoculations (representing 0 days post-inoculation, dpi) and at disease stages representing initial chlorotic flecks (2 dpi), advanced chlorotic flecks (5 dpi), lesions (7 dpi), and mature lesions (13 dpi, Figure 1). An additional sample of maize leaves inoculated with *E. turcicum* race 23N was collected at 13 dpi, which showed extensive damage to leaf tips and is from here on referred to as the “severe lesion” (SL). The transcriptome of a South African race 13N isolate grown *in vitro* was also sequenced to allow for comparisons between *in planta* and *in vitro* conditions and to identify *in planta*-specific transcripts, which may be more likely to be involved in pathogenicity. Single conidia of isolate 103 (race 13N) were obtained from diseased leaf material collected during the maize inoculation trial and grown on potato dextrose agar for 2 weeks before material was harvested for RNA sequencing.

### RNA Extraction and Sequencing

RNA was extracted from five technical replicates per biological replicate for *in planta* and *in vitro* samples by flash freezing leaf or fungal material (0.1 g) in liquid nitrogen and grinding the samples using a mortar and pestle. Total RNA was extracted from frozen material using the Qiazol lysis reagent (Qiagen, Limburg, Netherlands) according to the manufacturer's instructions. Genomic DNA contamination was removed with RNase-free DNase I (Qiagen) and extracted RNA purified using the RNeasy® Mini Kit (Qiagen) according to the manufacturer's instructions. RNA was eluted into a final volume of 30 μl with nuclease-free water. RNA concentration and purity was estimated with the Nanodrop® 2000 spectrophotometer (Thermo Fisher Scientific, Waltham, United States), and the quality of extracted RNA was visualized with formaldehyde gel electrophoresis (Bryant and Manning, 2000).

Before sequencing, three biological replicates per time point were pooled in 15 μg quantities of each replicate to obtain 45 μg of RNA per time point. The pooled RNA samples were analyzed on the 2100 Bioanalyzer (Agilent, California, United States) to ensure that samples were of adequate quality and quantity for transcriptome sequencing. In total, 12 RNA samples (13N_*in vitro*, 13N_0dpi, 13N_2dpi, 13N_5dpi, 13N_7dpi, 13N_13dpi, 23N_0dpi, 23N_2dpi, 23N_5dpi, 23N_7dpi, 23N_13dpi, and 23N_SL) were sent to the Beijing Genomics Institute (BGI; Shenzhen; China, RRID:SCR_011114) for library construction and strand-specific sequencing of 90 bp reads on the Illumina HiSeq 2500 platform (Illumina, California, United States, Illumina HiSeq 2500 System, RRID:SCR_016383). The raw RNAseq data have been deposited in the National Center for Biotechnology Information (NCBI) Short Read Archive (accession number PRJNA560644).

### Transcriptome Mapping and Quantification

Raw Illumina reads were quality trimmed and filtered to remove low-quality reads and adaptors using Trimmomatic v. 0.33 (Bolger et al., 2014) (Trimmomatic, RRID:SCR_011848) with the following settings: “ILLUMINACLIP = Yes, TruSeq3 (paired-end for MiSeq and HiSeq), HEADCROP = 10, MINLEN = 90.” The reference race 23N *E. turcicum* Et28A (Ohm et al., 2012; Condon et al., 2013) and *Zea mays* B73 RefGen_v3 (http://www.maizegdb.org/assembly/) genomes were concatenated, and filtered reads were aligned to the concatenated genome with Tophat v. 2.0.13 (Trapnell et al., 2009) using the following parameters: “mean inner distance between mate pairs = 170 bp, max realign edit distance = 0, library type = FR first strand.”

Read counts were determined with feature Counts (Liao et al., 2014) (featureCounts, RRID:SCR_012919) using the following command: “featureCounts –primary –p -a Settu1_GeneCatalog_genes_20110305.gff -o outputfile acceptedhits.bam.” The Bioconductor edgeR v. 3.12.1 package (Robinson and Smyth, 2008; Robinson and Oshlack, 2010; Robinson et al., 2010) (edgeR, RRID:SCR_012802) was used to remove genes that had fewer than one read per million mapped in at least one dataset. This package was also applied to normalize data. A multidimensional scatter (MDS) plot was constructed in edgeR using read counts of the genes mapped to the *E. turcicum* genome to visualize the similarity between datasets (Supplementary Figure 1). Datasets were normalized using the trimmed mean of *M* values (TMM) method implemented in edgeR (Robinson et al., 2010) (edgeR, RRID:SCR_012802). Heatmaps were drawn in the R package, pheatmap (Kolde, 2015).

### Fungal Quantification

A modified small-scale hexadecyltrimethylammonium bromide (CTAB) method described previously (Stewart and Via, 1993) was used to extract DNA from flash-frozen plant material collected for transcriptome sequencing. Fungal material was quantified using an *E. turcicum*-specific *cpr1* gene primer set and normalized relative to the amount of maize material estimated using the *gst3* gene primer set (Langenhoven et al., under review). The method developed and validated by Langenhoven et al. (under review) was based on the fungal quantification method developed for *Cercospora zeina* (Korsman et al., 2012). Quantities of *E. turcicum* and maize were extrapolated from standard curve graphs, and fungal quantification was determined as nanogram *E. turcicum* DNA per microgram maize DNA. Tests to detect significant differences were conducted using a one-way ANOVA and the Tukey multiple pairwise comparison at a 95% confidence interval in R (R Core Team, 2017) (R Project for Statistical Computing, RRID:SCR_001905) of log-transformed fungal quantities. The correlation between the percentage of reads mapped and log-transformed fungal quantities were investigated using the Spearman correlation method in R (R Core Team, 2017) (R Project for Statistical Computing, RRID:SCR_001905). A scatter plot was constructed to visualize the correlation between data using the “ggpubr” v 0.2 package in R (R Core Team, 2017) (R Project for Statistical Computing, RRID:SCR_001905).

### Functional Annotation

Annotations of the *E. turcicum* Et28A genome (race 23N) based on gene ontology (GO), InterProScan domains, Kyoto Encyclopedia of Genes and Genomes (KEGG), and EuKaryotic Orthologous Groups (KOG) were conducted previously (Ohm et al., 2012; Condon et al., 2013) and are available for download (http://genome.jgi.doe.gov/Settu1/Settu1.home.html). Genes predicted to be secreted as well as those annotated as carbohydrate-active enzymes (CAZymes) or involved in secondary metabolite biosynthesis are also available for download from the same source. The R packages GSEABase (Morgan et al., 2017) and GOStats (Falcon and Gentleman, 2007) were used to identify fungal overrepresented GO terms using the standard hypergeometric test at a significance level of 0.05. Overrepresented GO terms were subsequently grouped into high-level summaries using the online tool GOSlimViewer (McCarthy et al., 2006) (GOSlimViewer, RRID:SCR_005665). Cell wall degrading enzymes were identified from CAZymes by considering functional annotations and performing BLASTp analysis against the Plant Cell Wall-Degrading Enzyme database (Choi et al., 2013) using the following parameters: expect (*e*–) value <1 × 10^−5^ and percent similarity >40%. The Joint Genome Institute (JGI) database (https://mycocosm.jgi.doe.gov/Settu1/Settu1.home.html) was queried to identify genes involved in β-oxidation of lipids and fatty acids and the glyoxylate cycle.

### Identification of Candidate Effectors

Secreted proteins were previously identified from the *E. turcicum* genome (Ohm et al., 2012; Condon et al., 2013) and were downloaded from the Joint Genome Initiative website (http://genome.jgi.doe.gov/Settu1/Settu1.home.html) These protein sequences were investigated to identify candidate secreted effectors based on three categories: (1) protein characteristics, (2) evidence of expression, and (3) genome annotations or similarity to known proteins. Protein characteristics investigated were presence of a signal peptide, absence of a transmembrane domain, protein size, and cysteine content. The presence of a signal peptide was confirmed with SignalP (Petersen et al., 2011) (SignalP, RRID:SCR_015644) and TMHMM v2.0 (Sonnhammer et al., 1998) (TMHMM Server, RRID:SCR_014935) was used to detect transmembrane domains. A protein was labeled as a putative transmembrane protein if it contained more than two transmembrane domains in total, or at least one domain after the first 60 amino acids (Sperschneider et al., 2016). Since the exact size limit on effectors is still unclear, 350 aa was chosen as the limit of candidate effector size, and larger proteins were removed. Proteins containing less than two cysteine residues were also removed.

Proteins were considered to show evidence of expression if they contained read counts values >2 (based on transcriptome sequencing) in at least one dataset. Candidates were investigated for similarities to proteins with known roles in pathogenicity by performing BLASTp analysis on the Pathogen–Host Interactions database (PHI-base, RRID:SCR_003331) (Urban et al., 2017). BLASTp analysis was also performed against the non-redundant NCBI protein database using DIAMOND (Buchfink et al., 2015). Obtained hits from BLASTp searches against PHI-base and NCBI database with an *e*-value of <1 × 10^−5^ and similarity >40% were considered significant. In addition, similarity of *E. turcicum* proteins to secreted in xylem (SIX) proteins were investigated by querying the NCBI database (NCBI, RRID:SCR_006472) for proteins with the keyword “secreted in xylem SIX” (date search was performed: 18 April 2019). Gene names were manually investigated to identify search results that were not annotated as secreted in xylem. The protein sequences of all available *SIX* gene sequences were downloaded, and BLASTp analysis was performed against the *E. turcicum* Et28A proteome with DIAMOND (DIAMOND, RRID:SCR_009457) using an *e*-value cut-off of 1 × 10^−5^ and the parameter “more sensitive” (Buchfink et al., 2015). Heatmaps were constructed in pheatmap (Kolde, 2015) (pheatmap, RRID:SCR_016418), a package in R (R Core Team, 2017) (R Project for Statistical Computing, RRID:SCR_001905).

### Race Comparisons

Races were compared by scoring read count values as 1 (read count value >2) or 0 (read count value <2) across time points 2–13 dpi. Two *in planta* groups were subsequently created, which consisted of presence/absence per protein for 13N_2dpi, 13N_5dpi, 13N_7dpi, and 13N_13dpi as well as 23N_2dpi, 23N_5dpi, 23N_7dpi, 23N_13dpi, and 23N_SL—from here on referred to as 13N_*in planta* and 23N_*in planta*, respectively. Presence/absence was also scored for the 13N_*in vitro* data. Venn diagrams were constructed to compare expression between the 13N_*in vitro* data and the 13N_*in planta* group, as well as between the *in planta* groups. Proteins with expression in only one group or dataset or shared between groups and the *in vitro* dataset were submitted for BLASTp analysis against the non-redundant protein database with DIAMOND (Buchfink et al., 2015). Parameters were set to return the 20 most significant hits per query, and an *e*-value cut-off of 1 × 10^−5^ was used. In addition, putative functions of proteins were assigned based on InterProScan domains and KEGG and KOG annotations of the *E. turcicum* Et28A genome conducted previously (Ohm et al., 2012; Condon et al., 2013).

### Quantitative RT-PCR Analysis

Complementary DNA (cDNA) was synthesized from RNA using the High Capacity RNA-to-cDNA™ Kit (Thermo Fisher Scientific, Waltham, United States) according to the manufacturer's instructions. Synthesis of cDNA from biological replicates was performed separately and not in a pool as for transcriptome sequencing. Primer sets were designed for reverse transcriptase PCR (RT-PCR) from the *E. turcicum* Et28A v. 1.0 genome based on open reading frames (Supplementary Table 1). The following conditions were used to amplify fragments of each candidate effector gene from cDNA as templates for standard curves: 12.5 μl Amplicon Taq 1.1 Master Mix, 1.6 μM of each primer, and 30 ng DNA in a final volume of 25 μl. The initial denaturation step was at 95°C for 3 min, followed by 30 cycles of 95°C for 15 s, 61°C for 15 s, and 72°C for 15 s, with a final extension step of 72°C for 40 min. Amplicons were purified using Sephadex G50® columns.

Reference and target gene amplicons were cloned into the pJET1.2/blunt vector to use as DNA templates for standard curves for RT quantitative PCR (RT-qPCR). Ligation reactions were performed using the CloneJET PCR Cloning Kit (Thermo Fischer Scientific, Waltham, United States) according to the manufacturer's instructions. Purified amplicons were cloned into *Escherichia coli* DH5α using the heat shock method (Froger and Hall, 2007). Competent cells were prepared using the calcium chloride protocol (Holsters et al., 1978). Putative transformants were investigated for the presence of the insert in a colony PCR using 6.25 μl Amplicon Taq 1.1 Master Mix and 1.6 μM of each M13 primer in a total volume of 12.5 μl. Cycling conditions were the same as described above. Transformants containing the insert were cultured and plasmid DNA extracted using the Zyppy™ Plasmid Miniprep Kit (Zymo Research, Irvine, United States) as per the manufacturer's instructions.

Reverse transcriptase quantitative PCR reactions were performed for *E. turcicum* candidate effector genes and three reference genes, namely, *40S ribosomal protein* (*40S*, ProtID 168532), *elongation factor 1-*α (*EF1-*α, ProtID 36922), and *glyceraldehyde-3-phosphate dehydrogenase* (*GAPDH*, ProtID 183842) using the Bio-Rad CFX96 Touch™ Real-Time PCR Detection System. Reactions were set up in a total volume of 11 μl consisting of 5 μl RealQ Plus 2x Master Mix Green without ROX™ (Amplicon, Brighton, United Kingdom), 0.5 μM of each primer, 1 μl cDNA template, and sterile, distilled water. The cycling conditions were as follows: 95°C for 10 min, 45 cycles of 95°C for 10 s, 60°C for 15 s, and 72°C for 10 s. A standard curve was constructed for each gene (from starting concentrations ranging from 250.2 to 712 ng/μl) using serial dilutions of plasmids at 1 × 10^−2^-1 × 10^−6^.

The melt curve analysis of each gene was performed in the software package Bio-Rad CFX Manager™ to ensure that only a single cDNA product was produced and that primer dimers were absent. A product from each RT-qPCR experiment was purified (using the Sephadex® G50 protocol) and sequenced using 1 μl BigDye, 4 μl purified product, and 1.6 μM primer in a volume of 10 μl. Products were purified using the Sephadex® G50 spin columns and submitted for Sanger sequencing to confirm that the correct fragment was produced. RT-qPCR results were analyzed in qBASE^PLUS^ (Biogazelle, Zwijnaarde, Belgium).

Significant differences in gene expression between datasets were detected using a one-way ANOVA (ANOVA, RRID:SCR_002427), and the Tukey multiple pairwise comparison was conducted to identify significant differences between datasets at a 95% confidence interval. Analyses were conducted in R (R Core Team, 2017) (R Project for Statistical Computing, RRID:SCR_001905) using log-transformed calibrated normalized relative quantity (CNRQ) values. A constant value of 1 was added to all values to obtain positive log values. The null hypothesis tested was that no significant differences existed between datasets. At least two biological replicates were available for analyses, except for the 0 dpi time point, as no fungal transcripts were detected and this point was removed from the ANOVA (ANOVA, RRID:SCR_002427).

### Effector Sequencing

Twenty *E. turcicum* isolates from a previous population genetic study of *E. turcicum* were selected for sequencing based on genetic differences (Nieuwoudt et al., 2018). Five isolates were selected from each host (maize and sorghum) and location (Delmas and Greytown, South Africa) to represent a diverse set of isolates. DNA was extracted from pure cultures after 4–7 days growth using the Zymo Research Fungal/Bacterial DNA extraction kit (Zymo Research, Irvine, United States) as per the manufacturer's instructions, with extension of the vortex time (45 min rather than the 5 min as suggested). Sequences of each candidate effector from the sequenced *E. turcicum* Et28A v. 1.0 (race 23N, http://genome.jgi.doe.gov/Settu1/Settu1.home.html) and NY001 v. 2.0 (race 1, https://mycocosm.jgi.doe.gov/Settur3/Settur3.info.html) genomes were included to compare sequence variation among isolates from different continents. Candidate effector sequences from an *E. turcicum* isolate (Et73), which has been shown to be specific to sorghum, were also included (Langenhoven et al., under review).

Primers were designed to amplify the full sequences as well as flanking regions of two candidate effectors (Supplementary Table 1) based on the genome sequence of *E. turcicum* Et28A v.1.0 (Ohm et al., 2012; Condon et al., 2013). Conditions to amplify candidate effector gene sequences were as follows: 12.5 μl Amplicon Taq 1.1 Master Mix, 0.48 μM of each primer, and 30 ng DNA in a final volume of 25 μl using the same cycling conditions as described above. Amplicons were purified using the Sephadex® G50 spin columns and sequenced. Sequences were aligned using MUSCLE (Edgar, 2004) (MUSCLE, RRID:SCR_011812) in MEGA v.7.0.26 (Kumar et al., 2016) (MEGA Software, RRID:SCR_000667).

Phylogenetic trees depicting evolutionary relationships among the amino acid sequences of the candidate effectors sequenced during this study were inferred by maximum likelihood using the package PHANGORN in R v. 3.4.0 (Schliep, 2011). The optimal model of amino acid substitution was inferred using the “model test” function in the same package. The phylogenetic trees were drawn using 1,000 bootstraps, the optimal model of amino acid substitution and nearest-neighbor interchange to improve the likelihood of the tree. The haplotype network was drawn from nucleotide sequences using the median-joining method and an epsilon value of 0 in POPART v. 1.7 (Leigh and Bryant, 2015). Tajima's *D* test was performed in pegas, a package in R (Paradis, 2010), to determine if candidate effectors are undergoing positive selection.

## Results

### Disease Progression and Transcriptome Sequencing of *Zea mays* Seedlings Infected With *Exserohilum turcicum*

We applied dual RNA sequencing of pooled biological replicates to conduct genome-wide expression profiling of *E. turcicum* during infection of maize at five time points. Light, chlorotic flecks were observed at 2 dpi, which became more numerous at 5 dpi (Figures 1A,B). Tan-colored lesions were observed at 7 dpi, which enlarged and became gray in color at 13 dpi. At 13 dpi, maize seedlings displayed lesions without damage to leaf tips as well lesions with significant damage to leaf tips (referred to as severe lesions). Similar symptoms were observed for both races, as expected, since susceptible maize line Va26 lacks *Ht* genes (Figures 1A,B). For each time point, 15–17.5 million paired-end reads were produced (Table 1). In addition, 15.4 million paired-end RNAseq reads were generated from an *in vitro* grown race 13N *E. turcicum* culture (Table 1). Expression profiles of the 11,702 genes predicted from the *E. turcicum* Et28A genome (Ohm et al., 2012; Condon et al., 2013) revealed evidence of expression for 70% of genes *in vitro* (normalized expression values >2) and 86.8% during *Z. mays* infection (Supplementary Table 2). A low percentage of reads that mapped to the *E. turcicum* genome were detected before inoculation in the 13N_0dpi (0.0015%) and 23N_0dpi (0.0019%) datasets. The possibility that a small number of maize genes may have mapped to the fungal genome, or that reads mapped to endophytes present in maize, cannot be excluded. However, we do not believe that these results have a significant impact on our main findings as the RNAseq data was used for gene discovery and not differential expression. In this study, a dataset was considered to be either the read count values for the *in vitro* grown isolate or values for isolates of race 13N or 23N at a particular *in planta* time point.

**Table 1:** Overview of transcriptomic sequencing data collected for a time course experiment performed on a race 13N and a race 23N *Exserohilum turcicum* isolate.

**Dataset**	**No. of total reads (millions)**	**Et28A:B73v3^a^**		**Subset of reads mapping to Et28A^b^**	
		**No. of reads mapped (millions)**	**% of reads mapped**	**No. of reads mapped (millions)**	**% of reads mapped**
13N_*in vitro*	15.4	12.73	82.4	12.73	82.4
13N_0dpi	16.47	13.32	79.4	0.001	0.007
13N_2dpi	15.64	12.83	81	0.005	0.035
13N_5dpi	15.13	12.37	80.8	0.007	0.046
13N_7dpi	16.29	13.23	80.3	0.057	0.353
13N_13dpi	15.03	11.93	78.5	2.37	15.8
23N_0dpi	15.53	12.69	80.4	0.001	0.007
23N_2dpi	15.12	12.35	80.8	0.002	0.015
23N_5dpi	17.53	14.35	80.9	0.017	0.097
23N_7dpi	15.2	12.47	81	0.051	0.336
23N_13dpi	15.95	12.45	79.3	3.02	19.5
23N_SL	15.68	10.07	64.2	2.87	18.3

a *Total reads mapping to concatenated E. turcicum and maize genomes*.

b *Subset to reads that mapped to E. turcicum genome*.

The percentage of transcripts mapped at each time point showed the same trend (low at 0–7 dpi, increasing at 13 dpi) for both races. *E. turcicum* genomic DNA (gDNA) quantity measured by qPCR (as a proxy for fungal biomass) was significantly higher at 13 dpi as compared to 0–7dpi with no differences detected between 13N_13dpi and 23N_13dpi (Figure 1C). There was a significant positive correlation between the percentage of reads mapped and log-transformed fungal gDNA content (Figure 1D). These results indicate extensive colonization of maize leaves by *E. turcicum* between days 7 and 13.

### Functional Annotation

#### Overrepresented Gene Ontology Terms

First, we conducted GO enrichment analysis of expressed *E. turcicum* genes at each time point to determine whether specific biological processed or functions were enriched over the time course. Overrepresented GO terms related to biological process were identified in 10 of the 12 datasets (13N_*in vitro*, 13N_0dpi to 13N_13dpi, and 23N_0dpi to 23N_7dpi) and in 11 datasets (13N_*in vitro*, 13N_0dpi to 13N_13dpi, and 23N_0dpi to 23N_13dpi) for the molecular function GO terms (Supplementary Figure 2, Supplementary Table 3). Two GO terms related to fungal pathogenicity, namely, peptidase activity and carbohydrate metabolic process, were significantly overrepresented in the high-level summaries. This led us to next investigate the peptidases and cell wall degrading enzymes encoded in the *E. turcicum* genome that were expressed during maize infection.

#### Protein Degradation

**Figure 2:**
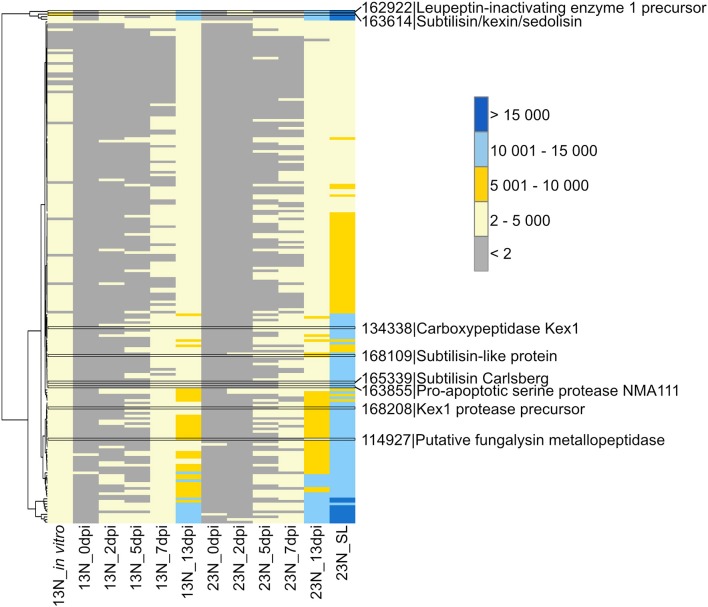
Transcriptional profiling of *Exserohilum turcicum* peptidases. Peptidases were previously identified from the *E. turcicum* Et28A genome (Ohm et al., 2012). Peptidases of interest are highlighted by black bars. Row names are given as protein identifier|description. The scale bar represents read count values.

A total of 198 peptidases belonging to the aspartyl-, cysteine-, serine-, gamma-glutamyl-, glutamate-, metallo-, serine-, and threonine protease classes were previously identified from the genome (Ohm et al., 2012; Condon et al., 2013) and showed evidence of expression in this study (Supplementary Table 4). Two of the peptidases with the highest expression levels were a peptidase M28 protein (ProtID 162699, Figure 2) similar to leupeptin-inhibiting protein 1 and a protein with similarity to peptidase S8/S53 subtilisin/kexin/sedolisin from *Macrophomina phaseolina* (ProtID 163614). A protein (ProtID 114927) showed similarity to a putative fungalysin metallopeptidase from *Colletotrichum sublineola*. A protein with similarity to Kex1 protease precursor from an unidentified species of the Ascomycete genus *Pyrenochaeta* was identified (ProtID 168208) as well as a protein (ProtID 134338) with similarity to the pheromone processing carboxypeptidase Kex1 from *Leptosphaeria maculans*. Two proteins with similarity to subtilisins were identified, one to a subtilisin-like protein from *Glonium stellatum* (a mycorrhizal fungus of the class *Dothideomycetes*, ProtID 168109) and another subtilisin Carlsberg from the pathogen, *Colletotrichum nymphaeae* (ProtID 165339). A protein with similarity to a proapoptotic serine protease NMA111 from an unidentified *Pyrenochaeta* species was also identified (ProtID 163855).

#### Plant Cell Wall Degradation

**Figure 3:**
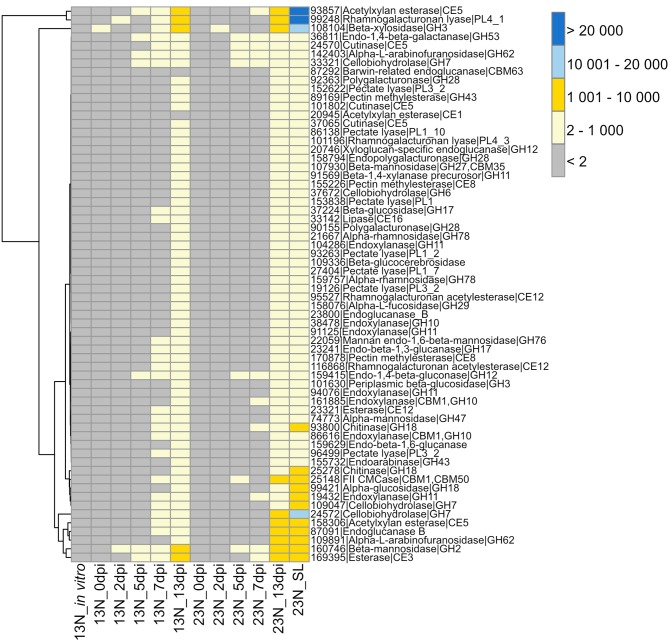
Transcriptional profiles of genes expressed in *Exserohilum turcicum* putatively involved in cell wall hydrolysis. Heatmap showing read count values for 64 cell wall degrading enzymes (CWDEs) observed *in planta* but not *in vitro*. Putative CWDEs were identified by performing BLASTp analysis of carbohydrate active enzymes (Ohm et al., 2012) against the Plant Cell Wall-Degrading Enzymes database (Choi et al., 2013). Only hits with an expect value of <1 × 10^−5^ and similarity of more than 40% were considered significant. Row names are given as “protein ID|sequence description|CAZyme ID.” The scale bar represents read count values.

The GO term “carbohydrate metabolic process” was noted to be unique to the *in planta* datasets. We were particularly interested in the CWDE subset of the carbohydrate active enzymes (CAZYmes), since cell wall degradation is expected to be a major component of the necrotrophic phase of *E. turcicum* infection. A total of 478 unique CAZYmes were previously identified from the genome (Ohm et al., 2012; Condon et al., 2013). Of these, 393 show evidence of expression and were specifically examined for a putative role in cell wall hydrolysis and degradation. A total of 175 genes involved in cell wall degradation were identified (Supplementary Table 5) of which 64 CWDEs were expressed *in planta* but not *in vitro* (Figure 3). Of these, 36 were significantly expressed (read count >2) at 13 dpi only. The list included mannosidase, endoxylanase, glucosidase, cutinase, as well as pectin- and pectate lyases.

#### Secondary Metabolite Production

**Figure 4:**
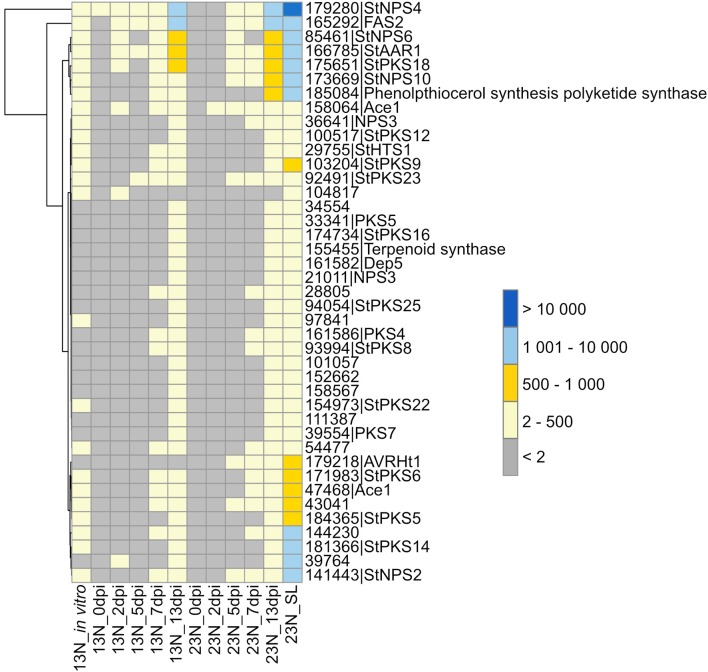
Expression profiles of genes involved in secondary metabolite synthesis. Secondary metabolites previously identified from the *E. turcicum* Et28A genome (Ohm et al., 2012) were investigated for similarity to known pathogenicity determinants on the Pathogen–Host Interactions database (Urban et al., 2017). Hits with an expect value of <1 × 10^−5^ and a similarity percentage >40 were considered as significant. Row names are given as “protein ID|sequence description.” The scale bar represents read count values.

Plant infection by fungi, specifically during the necrotrophic phase, is often associated with secondary metabolite production, some of which are phytotoxins. Therefore, we queried our datasets for expression of *E. turcicum* genes encoding secondary metabolite biosynthesis enzymes. Out of the 49 genes involved in secondary metabolite production previously identified from the genome of *E. turcicum*, 41 showed evidence of expression in this study (read count >2, Figure 4, Supplementary Table 6). Among the most highly expressed secondary metabolite biosynthetic enzymes were StNPS4 (ProtID 179280) and a fatty acid synthase (FAS2, ProtID 165292). Five secondary metabolite enzymes had significant hits (*e*-value < 1 × 10^−5^, % identity >40%) to proteins on PHI-Base previously characterized to result in loss of pathogenicity or reduced pathogenicity during knockout analyses. Two proteins previously annotated as non-ribosomal peptide synthetases from *E. turcicum*, StNPS6 (ProtID 85461) and StNPS2 (ProtID 141443), matched proteins from *Cochliobolus sativus* (causal agent of spot blotch on barley) and *Cochliobolus heterostrophus (*causal agent of southern corn leaf blight), respectively. An alpha-aminoadipate reductase (AAR, protID166785) from *E. turcicum* matched a protein from *C. sativus*, which resulted in loss of pathogenicity during knockout experiments (Leng and Zhong, 2012). Two *E. turcicum* proteins (ProtIDs 47468, 158064) had significant hits to the same hybrid PKS:NPS, namely, ACE1 from *Magnaporthe oryzae*.

Two *E. turcicum* proteins with significant hits to previously characterized toxin synthases were detected. The protein StNPS3 (ProtID 36641) had a significant hit to tentoxin synthase (TES) from *P. tritici-repentis*, but expression was low. StNPS10 (ProtID 173669) produced a significant hit to the enzyme synthesizing the antibiotic tyrocidine (tyrocidine synthetase 1) from the *Dothideomycete* plant pathogen, *Stemphylium lycopersici*. A protein with similarity to the HC-toxin synthase (*HTS1*, ProtID 29755) was detected although expression levels were low (Figure 4, Supplementary Table 6). Orthologs of the *TOX* genes that form the *TOX2* locus together with *HTS1* have previously been identified in *E. turcicum*, and read count values suggested that all *E. turcicum TOX* orthologs were expressed, with high expression of the *TOXC* (ProtID 165293) and *TOXG* orthologs (ProtID 168012, Supplementary Table 6).

#### The β-Oxidation Pathway

**Figure 5:**
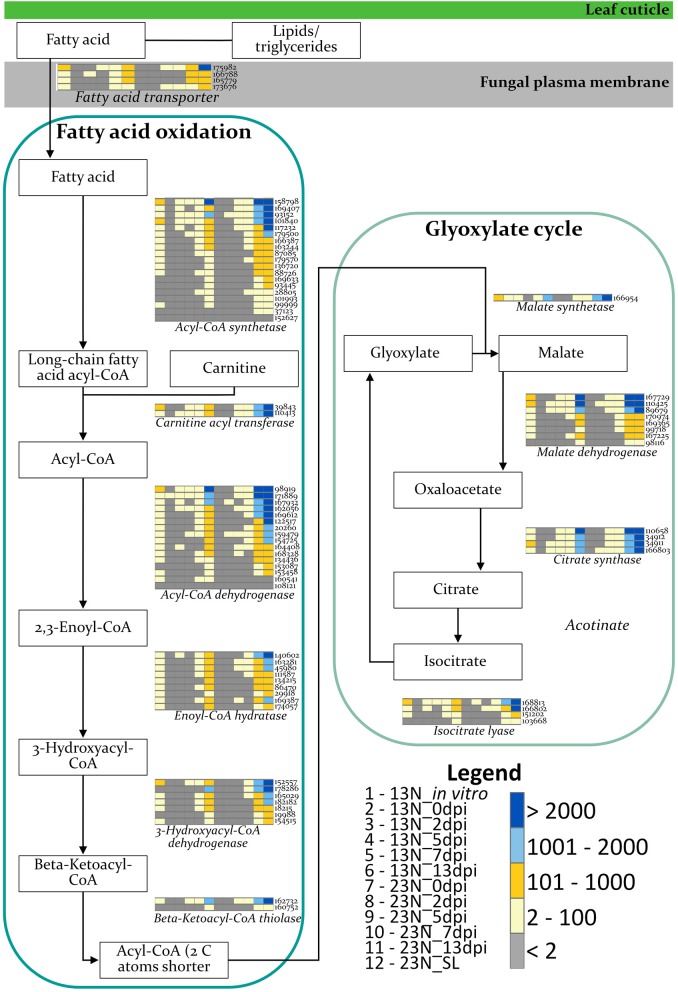
Expression profiles of *E. turcicum* genes involved in the β-oxidation pathway. Expression of enzymes involved in fatty acid oxidation and glyoxylate cycles (Rudd et al., 2015; Zhang et al., 2018) shows that *E. turcicum* utilizes host resources during early stages of infection. The scale bar represents read count values. Columns are expression profiles for a gene as given by the protein identifier.

The expression of genes involved in β-oxidation was investigated to identify the nutrient source used by *E. turcicum* during host colonization (Figure 5). Genes involved in β-oxidation and fatty acid metabolism were expressed across all time points, with the exception of genes involved in the synthesis of enoyl-CoA hydratase and 3-hydroxyacyl-CoA dehydrogenase (Figure 5). The read counts of genes coding for enoyl-CoA hydratase indicated expression of an ortholog in 13N_2dpi but absent in 13N_5dpi, 23N_2dpi, and 23N_7dpi. Expression of other paralogs of the same gene was observed in 23N_5dpi, but not 13N_2dpi, 13N_5 dpi, and 23N_2dpi. Expression for 3-hydroxyacyl-CoA dehydrogenase was only obtained for one paralog at 13N_5dpi.

### Candidate Effector Proteins of *E. turcicum*

Several lines of evidence were followed to identify *E. turcicum* secreted proteins that could be candidate effectors with a role in pathogenicity, namely (i) protein characteristics, (ii) sequence similarity to known effectors, (iii) virulence function shown for a similar protein in another fungal phytopathogen, and (iv) expression *in planta*. A total of 1,388 secreted proteins previously identified from the race 23N *E. turcicum* Et28A genome were downloaded and queried to identify candidate effectors (Ohm et al., 2012; Condon et al., 2013). Of these, 1,186 had more than 1 read per million mapped in at least one dataset in our study. A total of 351 proteins met the criteria to be classified as candidate effectors, of which 346 showed evidence of expression (read count value >2) in at least one dataset (Supplementary Table 7).

Similarity searches of candidate effectors against PHI-BLAST revealed significant hits to previously characterized effectors or effector candidates (8 hits) or to proteins with an increased (3), reduced (15), or mixed effect (3) on virulence when knocked out (Table 2). Known effectors included Ecp6 (ProtID 136414), which was previously characterized in *E. turcicum* (Xue et al., 2013). One protein (ProtID 30080) had a significant hit to the secreted in xylem 5 (SIX5) effector from *Fusarium oxysporum*. Another protein (ProtID 20746) exhibited similarity to the XEG1 protein from *Phytophthora sojae*. Three proteins (ProtIDs 154392, 164382, and 28054) had significant hits to the MoCDIP4 effector from *M. oryzae*. A hit to BEC1019 (ProtID 91360), a candidate effector from *Blumeria graminis*, was identified. A protein (ProtID 30084) similar to the secreted lipase effector FGL1 from *Fusarium graminearum* (Blümke et al., 2014) was detected.

**Table 2:** Effectors and pathogenicity determinants from the *Exserohilum turcicum* Et28A genome with matches in the PHI database and that were expressed in race 13N and/or race 23N *Exserohilum turcicum*.

**ProtID^a^**	**Protein name^b^**	**Sequence description**	**PHI accession**	**Effect on virulence^c^**	***e*-value**	**%ID**	**Species to which best hit was identified**	**Expression^d^**
30080	SIX5	Effector	5286	Effector	8.6 × 10^−22^	42.31	*Fusarium oxysporum*	*In planta*
136414	Ecp6	Effector	5495/5543/5576	Effector	4.7 × 10^−44^	43.53	*Cladosporium fulvum*	All
19126	PELA	Pectate lyase, virulence factor	179	Reduced	1.5 × 10^−63^	44.26	*Nectria haematococca*	*In planta*
18525	MHP1	Hydrophobin	458	Reduced	1.7 × 10^−09^	49.06	*M. oryzae*	*In planta*
90318	MHP1	Hydrophobin	458	Reduced	8.1 × 10^−17^	43.16	*M. oryzae*	*In planta*
169551	FgVps29	Retromer core component	6093	Reduced	3.5 × 10^−89^	65.33	*F. graminearum*	All
24117	BcPIC5/BcFKBP12	Unknown|Rapamycin sensitivity	548/2305	Mixed	6.1 × 10^−21^	45.1	*Botrytis cinerea*	All
20746	XEG1	Effector	4980	Effector	8.2 × 10^−52^	43.26	*Phytophthora sojae*	*In planta*
28054	MoCDIP4	Effector	3216	Effector	1.0 × 10^−26^	41.11	*Magnaporthe oryzae*	*In planta*
154392	MoCDIP4	Effector	3216	Effector	1.6 × 10^−30^	40.65	*M. oryzae*	All
164382	MoCDIP4	Effector	3216	Effector	4.2 × 10^−92^	65.24	*M. oryzae*	All
30084	FGL1	Effector lipase, virulence factor	432/4212/4242	Reduced	7.2 × 10^−68^	43.69	*F. graminearum*	All
91360	BEC1019	Effector	2897	Reduced	3.1 × 10^−74^	41.85	*Blumeria graminis*	All
41216	NLP2	Necrosis and ethylene inducing like protein	2712	Reduced	1.8 × 10^−77^	58.96	*Verticillium dahliae*	All
27404	PELB	Pectate lyase, virulence factor	222	Reduced	2.2 × 10^−77^	50.76	*C. gloeosporioides*	*In planta*
96499	PELA	Pectate lyase, virulence factor	179	Reduced	1.1 × 10^−45^	43.3	*N. haematococca*	*In planta*
152622	PL1332	Pectate lyase coding gene	4845	Reduced	2.9 × 10^−80^	55.26	*Alternaria brassicicola*	23N_*in planta*
174636	endo-1-4-beta-xylanase precursor (GH10 family)	Endo-β-1,4-xylanase	2206	Reduced	5.8 × 10^−83^	43.6	*M. oryzae*	All
181799	Eng1	Secreted(1,3)-glucanase	6265	Reduced	8.9 × 10^−92^	49.18	*Histoplasma capsulatum*	All
167311	GAS2	Appressorial penetration	257	Reduced	4.9 × 10^−102^	55.13	*M. oryzae*	All
27270	GAS2	Appressorial penetration	257	Reduced	9.8 × 10^−90^	52.69	*M. oryzae*	*In planta*
168090	MGG_10510	Hypothetical	811	Reduced	2.2 × 10^−135^	62.82	*M. oryzae*	All
177420	Ppt1	Sfp-type 4′-phosphopantetheinyl transferase	4989	Reduced	0	89.91	*C. miyabeanus*	All
178168	RED3	Putative short chain dehydrogenase	2836	Reduced	2.5 × 10^−36^	47.29	*C. heterostrophus*	All
85317	MfCUT1	Cutinase	2383	Increased	1.9 × 10^−42^	47.65	*Monilinia fructicola*	All
88127	Hsp150p	Heat shock protein	5043	Increased	1.1 × 10^−26^	57.3	*Saccharomyces cerevisiae*	All
164814	SP1	Encoding SnodProt1	3221	Increased	5.7 × 10^−49^	60.31	*Parastagonospora nodorum*	All
155226	BCPME1	Pectin methylesterase	278/1028	Mixed	8.8 × 10^−95^	48.73	*B. cinerea*	*In planta*
153838	CcpelA	Pectate lyase	2476	Mixed	6.3 × 10^−54^	45.12	*C. coccodes*	*In planta*
166304	Ip	Succinate dehydrogenase subunit	822	Resistance to chemical	7.5 × 10^−178^	80.27	*Zymoseptoria tritici*	All

a *E. turcicum protein ID (based on the Et28A genome)*.

b *Name of protein in the Pathogen–Host Interactions database (PHI-base) (Urban et al., 2017) to which the respective E. turcicum protein is most similar. The sequence description and accession number of proteins in PHI-base are given. Best hits are based on the expect (e-) value and percentage identity (%ID)*.

c *Effect on virulence is reported for proteins on PHI-base, and was reduced, increased, or mixed, in which case both phenotypes were observed in different studies (as defined in PHI-base). In the case of previously characterized effectors, the term “effector” is assigned by PHI-base rather than virulence effect*.

d *Expression of candidate effectors and pathogenicity determinants are indicated as “All,” which includes expression in vitro and 13N _in planta and 23N_in planta datasets, “In planta,” which includes expression only in the 13N_in planta and 23N_in planta datasets or 23N_in planta, which indicates expression in these datasets only*.

Candidate effectors identified with similarity to proteins that resulted in reduced virulence when knocked out included cell wall degrading enzymes and proteins involved in appressorial penetration. Interestingly, a putative necrosis-and-ethylene inducing precursor protein was identified (ProtID 41216). A protein with similarity to Sfp-type 4′-phosphopantetheinyl (ProtID 177420) transferase was identified. Three significant hits were detected to proteins that resulted in increased virulence when overexpressed and included a cutinase from *Monolinia fructicola* (ProtID 85317), a heat shock protein from *Saccharomyces cerevisiae* (ProtID 88127) and SP1 from *Parastaganospora nodorum*, which shows high sequence homology to cerato-platanin (ProtID 164814), a phytotoxic protein of *Ceratocystis fimbriata* f. sp. *plantani*.

Homology searches against the NCBI database revealed eight additional candidates with putative annotations related to pathogenicity (Table 3). Four significant hits to known effectors were identified: ProtID 34559 showed similarity to the secreted in xylem 13 (SIX13) protein from *F. oxysporum*, ProtID 29144 was similar to the celpoo28 effector-like protein, and two proteins (ProtIDs 174473 and 184152) were identified with significant similarity to the biotrophy-associated protein 2 (BAS2). One cell death inducing protein (ProtID 25241) and the hypersensitive response inducing protein 1 from *Alternaria alternata* (ProtID 164162) was identified. One significant hit was identified as a chitin-binding protein of the *Dothideomycete* tomato pathogen, *S. lycopersici* (ProtID 135655). A significant hit to the PR1-like protein was obtained against *A. alternata* (ProtID 177800).

**Table 3:** Identification of candidate *Exserohilum turcicum* Et28A effector genes based on similarity to known proteins in the NCBI database.

**ProtID^a^**	**Description^b^**	**Accession**	***e*-value**	**%ID**	**Species to which best hit was identified**	**Expressed *in planta*^c^**
174473	Biotrophy-associated secreted protein 2 (BAS2)	OCT54266.1	1.20 × 10^−37^	49	*Cladophialophora carrionii*	All
184152	Biotrophy-associated secreted protein 2 (BAS2)	XP_018072866.1	2.30 × 10^−27^	72.3	*Phialocephala scopiformis*	All
25241	Cell death in tomato 1 (CDiT1)	OAL49902.1	4.80 × 10^−21^	62.8	*Pyrenochaeta* sp.	*In planta*
29144	Celp0028 effector like protein	KNG45831.1	8.70 × 10^−54^	46.6	*Stemphylium lycopersici*	*In planta*
135655	Chitin binding protein	KNG51028.1	3.20 × 10^−95^	63	*Stemphylium lycopersici*	All
164162	Hypersensitive response inducing protein 1	XP_018389270.1	7.80 × 10^−48^	56.1	*Alternaria alternata*	*In planta*
177800	PR-1-like protein	XP_018388239.1	1.10 × 10^−99^	78.4	*Alternaria alternata*	All
34559	Secreted in xylem 13 (SIX13), partial	ALQ80840.1	1.20 × 10^−14^	27.6	*Fusarium oxysporum* f. sp. *cubense*	*In planta*

a *E. turcicum protein ID (based on the Et28A genome)*.

b *Secreted proteins of <350 amino acids (aa) that do not contain transmembrane domains were further investigated for similarity to known effectors against the nonredundant NCBI protein database using DIAMOND (Buchfink et al., 2015). Best hits were identified based on the lowest expect value (e-value) and highest percentage identity (%ID)*.

c *Expression of candidate effectors and pathogenicity determinants are indicated as “All,” which includes expression in vitro and 13N_in planta and 23N_in planta datasets, or “In planta,” which includes expression only in the 13N_in planta and 23N_in planta datasets*.

An additional seven candidates contained annotations indicative of pathogenicity (Supplementary Table 7). Two proteins (ProtIDs 165307 and 165528) were annotated as containing “common in several fungal extracellular membrane proteins” (CFEM) domains. A protein (ProtID 34628) was identified, which contains a peptidoglycan-binding LysM domain, and another protein (ProtID 166607) had a chitin-binding domain. Three proteins with peptidase activity were identified, of which two are annotated as metalloproteases (ProtID 23005 and 30401) and one as a serine peptidase with trypsin activity (ProtID 93425).

A total of 558 proteins representing SIX1–SIX14 from different fungi were identified in the NCBI database by a keyword search (Supplementary Table 8). BLASTp analysis of these protein sequences against *E. turcicum* proteome yielded a total of 40 significant hits (Supplementary Table 8). Four *E. turcicum* proteins (ProtIDs 34559, 30080, 18972, and 24515, Supplementary Table 8) matched multiple proteins previously characterized as SIX13 or SIX5 from *F. oxysporum, Colletotrichum fructicola*, and *Neofusicoccum parvum*. The *E. turcicum* proteins most similar to SIX13 and SIX5 were ProtID 34559 and ProtID 30080, respectively (Supplementary Table 8). Although ProtID 34559 was the top hit to *F. oxysporum* SIX13, it had a low percentage identity and no functional annotation. Therefore, to gain further evidence that it might be a SIX13 ortholog, reciprocal BLASTp analysis was performed against the NCBI non-redundant protein database using the *F. oxysporum* f. sp. *cubense* SIX13 amino acid sequence (GenBank accession number ALQ80840.1) as input. Among the top 30 results were proteins previously identified as SIX 13 proteins from *F. oxysporum* as well as ProtID 34559 from *E. turcicum* (query coverage = 84%, *e*-value = 2 × 10^−16^, identity = 27%). Known SIX13 effectors identified from the NCBI database were aligned with *E. turcicum* Et28A ProtID 34559 (Supplementary Figure 3). Owing to the low percentage similarities of these proteins to the *F. oxysporum* sequences, these proteins were renamed as SIX13-like and SIX5-like.

**Figure 6:**
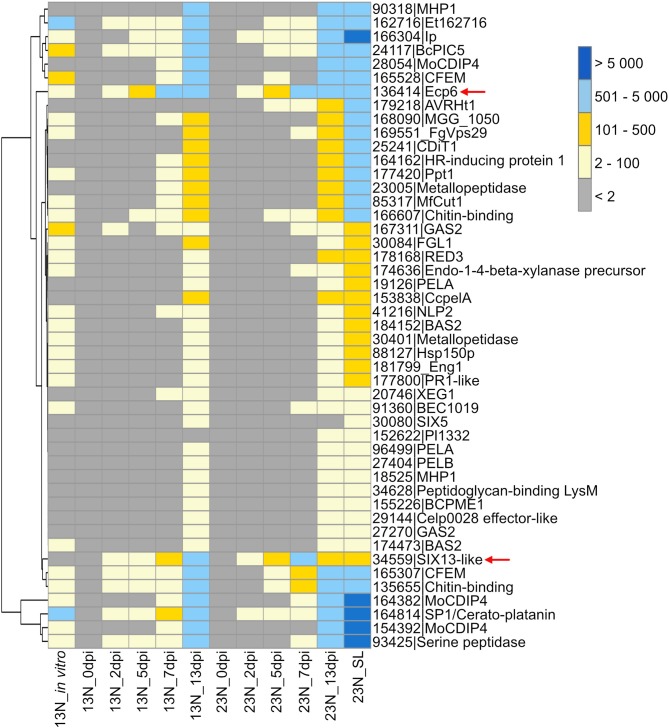
Transcriptional profiles of *Exserohilum turcicum* candidate secreted effector proteins similar to known effector proteins which affect virulence. A subset of candidate effectors was selected based on similarity to known proteins on the PHI-Base or NCBI database, annotations, or literature. The scale bar indicates the read count values. Row names are given as protein identifier|Gene name/description. Column names indicate the isolate race (13N or 23N) as well as days post-inoculation (dpi). Arrows indicate candidates selected for reverse transcriptase quantitative PCR (RT-qPCR) analysis.

A gene expression heatmap was generated of candidate *E. turcicum* effectors from this study that were annotated based on matches to *E. turcicum* Et28A secreted proteins, effectors in PHI-base, the NCBI database, or literature (Figure 6). The trend observed for all datasets was highest read count values for transcripts at 13 dpi, which is possibly due to the higher fungal reads mapped at this time point. The candidates with the highest expression values at 13 dpi were MoCDIP4 (ProtID 164382 and 154392), cerato-platanin (ProtID 164814), and the candidate annotated with trypsin activity (ProtID 93425). The candidate effectors showing the highest read counts at 2, 5, and 7 dpi included Ecp6, cerato-platanin, and ProtID 162716. The latter protein did not have a functional annotation, but it was also expressed earlier (at 2 dpi) in race 13N than race 23N (Figure 6). Ecp6 (ProtID 136414) and cerato-platanin were also expressed *in vitro*. Interestingly, *AVRHt1* showed no evidence of expression in the 13N_*in vitro* or in the 13N_*in planta* datasets but was detected in the 23N_*in planta* datasets. SIX5-like (ProtID 30080) was expressed at the latest time points *in planta*, whereas SIX13-like (ProtID 34559) showed expression at all time points in both races (Figure 6).

### Race Comparisons

**Figure 7:**
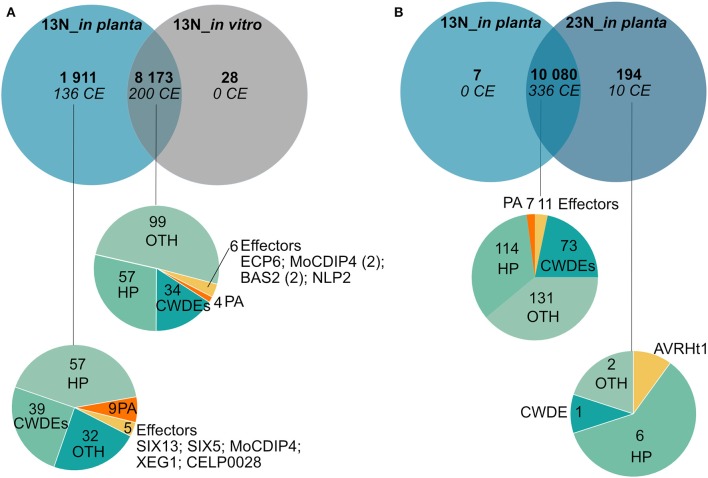
Comparison of genes expressed *in planta* and *in vitro* between a race 13N isolate and a race 23N *Exserohilum turcicum* isolate. Gene expression unique to a dataset was investigated by first clustering time points into two groups based on race, namely, 13N_*in planta* (all time points combined) and 23N_*in planta* (all time points combined). Thereafter, transcripts were denoted as either present (1) (read count value >2) or absent (0) (read count value <2) in each dataset. Similarly, presence/absence scores were obtained for the 13N_*in vitro* dataset. **(A)** Genes shared between the 13N_*in planta* group and 13N_*in vitro* database indicates that the majority of genes expressed are shared between isolates. **(B)** Comparison of gene expression between *in planta* 13N and 23N groups show limited genes unique to a group. Genes were assigned to classes and labeled with the following annotations: HP, hypothetical proteins; CWDE, cell wall degrading enzymes; CE, candidate effectors; PA, pathogenicity-associated proteins; or OTH, other (no role in pathogenicity).

Comparison of genes expressed in race 13N (all *in planta* time points vs. *in vitro*), as well as race 13N and 23N (all *in planta* time points) was carried out to identify race- or *in planta*-specific gene expression (Figure 7). Comparisons of the 13N *in planta* and *in vitro* conditions revealed that the majority of genes were shared between the datasets (8,173, Figure 7A, Supplementary Table 9). Of these, 200 were putatively identified as effectors. A total of 1,911 showed evidence of expression in the 13N_*in planta* group but not *in vitro* and included 136 putative effectors, which included five known effectors (SIX13-like, SIX5-like, MoCDIP4, XEG1, and CELP0028).

A total of 10,080 genes showed evidence of expression in both the 13N_*in planta* and 23N_*in planta* groups (Figure 7B, Supplementary Table 9). Of these, 336 were classified as putative effectors, of which 11 were similar to known effectors (including Ecp6). Seven genes were uniquely expressed in the 13N_*in planta* group, none of which were putatively identified as effectors or involved in pathogenicity. A total of 194 genes showed expression in the 23N_*in vitro* group only and included genes possibly involved in pathogenesis, cell wall hydrolysis, and secondary metabolite biosynthesis, as well as genes encoding transporter proteins, a hard surface induced and a defense-related protein (Supplementary Table 9).

Ten candidate effectors were uniquely expressed in the race 23N*_in planta* group and included *AVRHt1*. Mideros et al. (2018) reported a non-synonymous mutation in the *AVRHt1* (ProtID 179218), which distinguished race 1 and 23N isolates. Race 1 isolates encoded a “T” nucleotide and race 23N isolates a “C” allele. Transcriptome sequences generated during this study for each dataset revealed that the race 23N *E. turcicum* isolate encoded a “C” allele at the reported genomic location (scaffold 2: 3,549,698).

### RT-qPCR Validation of Candidate Effector Expression

The conserved effector *Ecp6* and candidate effector *SIX13-like* were chosen for expression validation. The RNAseq data had shown that Ecp6 was expressed *in vitro* and *in planta*. Candidate effector SIX13-like was not expressed *in vitro* in race 13N and was among the most highly expressed transcripts, with the highest expression at 2 dpi in race 23N and 5 and 7 dpi in both races.

**Figure 8:**
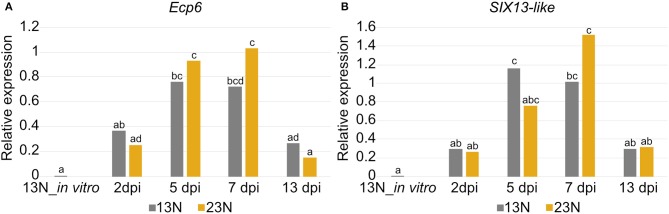
Expression analysis of two candidate effectors from *Exserohilum turcicum*. Real-time quantitative PCR (RT-qPCR) data are shown for *Ecp6* **(A)** and *SIX13-like* **(B)** from each *Exserohilum turcicum* race *in planta* at several time points, as well as *in vitro* (for race 13N). The *y*-axis units are the relative expression values (log-transformed mean calibrated normalized relative quantities). Analysis of molecular variance (ANOVA) and the Tukey multiple pairwise comparison was performed to identify pairwise differences in R (R Core Team, 2017) (R Project for Statistical Computing, RRID:SCR_001905). Different lowercase letters indicate significant differences between datasets. Significant pairwise differences were detected *in planta* for *Ecp6* **(A)** and *SIX13-like* **(B)**. The samples collected before inoculation (0 dpi) were excluded as no fungal transcripts were detected.

Significant differences in expression were detected between time points for *Ecp6* and *SIX13-like* (Figure 8). *In vitro* expression in race 13N was low for both candidate effectors. The expression of both *Ecp6* and *SIX13-like* peaked at 5 and 7 dpi as compared to 2 and 13 dpi. *Ecp6* expression was significantly greater at these mid-time points compared to the outer time points for race 23N, whereas *SIX13-like* expression was significantly greater at 5 dpi in 13N and 7 dpi in 23N compared to the outer time points in the corresponding race. The pattern of expression of each gene did not differ significantly between the two races. The identity of RT-qPCR products produced were confirmed with sequencing (Supplementary Figure 4).

### Sequencing of Candidate Effectors From Diverse *E. turcicum* Isolates

**Figure 9:**
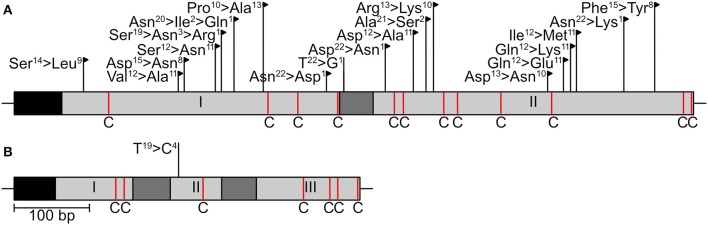
Sequence variation in candidate effector genes of *Exserohilum turcicum*. The sequences of two candidate effectors (**A** *SIX13-like*, **B** *SIX5-like*) were determined in 23 *E. turcicum* isolates from maize and sorghum. Black boxes indicate signal peptides, light gray blocks represent exons, and introns are shown as dark gray blocks. Cysteine residues are indicated as red lines with a “C.” Nucleotide substitutions that resulted in non-synonymous amino acid changes are indicated by black flags, while substitutions resulting in synonymous changes are indicated by black lines. The number of isolates representing each amino acid/nucleotide is given as a superscript number.

Two effector genes, *SIX13-like* and *SIX5-like*, were sequenced to identify host-specific differences among *E. turcicum* from maize and sorghum. A total of 22 polymorphisms were detected in the sequences of SIX13-like among the 20 *E. turcicum* isolates from maize and sorghum sequenced during this study, a sorghum-specific isolate (Et73, Langenhoven et al., under review), as well as genome sequences of Et28A and NY001 (Figure 9A). All SNPs detected in exon regions of *SIX13-like* resulted in non-synonymous amino acid changes, and no premature stop codons were detected. A SNP was detected in *SIX5-like* that resulted in a non-synonymous amino acid change (Figure 9B).

**Figure 10:**
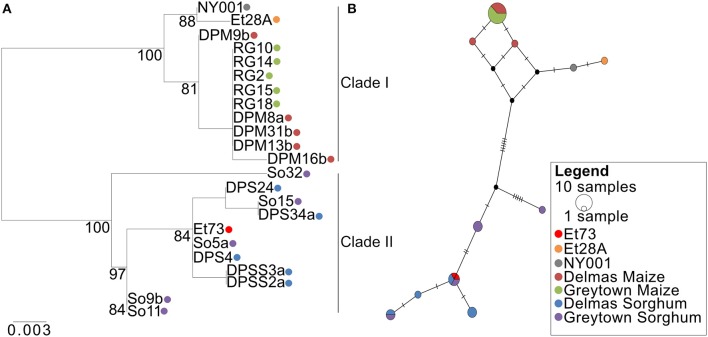
Phylogenetic relations of the *SIX13-like* effector among *Exserohilum turcicum* isolates from maize and sorghum shows host specificity. **(A)** A Maximum likelihood phylogenetic tree was drawn from 1,000 bootstrap repetitions and bootstrap values exceeding 75% are indicated in the graph. **(B)** Median-joining network of *SIX13-like* haplotypes based on nucleotide sequences. The size of the node represents shared haplotypes, and colors of the nodes are indicative of the host and location from where the isolates were collected. Black nodes represent missing or unsampled haplotypes. Bars represent mutational steps between haplotypes.

Alignment of the SIX13-like amino acid sequences showed that maize isolates were distinct from sorghum isolates with particular amino acids present in all or most isolates from a host (Supplementary Figure 5). This host specificity was borne out by maximum likelihood analysis that produced a phylogram with distinct maize and sorghum clades (Figure 10A). The optimal model of amino acid substitution used for this analysis was FLU, as determined by the lowest Akaike and Bayesian Information Criteria and highest log likelihood. The *E. turcicum* maize isolates from the United States (ET28A and NY001) formed a subclade of the South African maize isolates. The collection site in South Africa (Delmas or Greytown) did not influence the grouping of isolates (Figure 10A). The distinction between hosts was also visible from the haplotype network (Figure 10B) and showed a greater haplotype diversity among isolates from sorghum than maize. Tajima's *D* test was performed to determine if candidate effector *SIX13-like* is undergoing positive selection. Results indicated that the observed mutation rate was not significantly different from the null hypothesis of neutral selection.

## Discussion

At the start of this study, knowledge about *E. turcicum* genes expressed at different disease stages of host infection was lacking, despite the availability of the genome. Here, we provide a more detailed picture of the global transcriptome that is active in *E. turcicum* during maize seedling infection. Owing to the lack of sequenced biological replicates, it was not possible to study differential expression of genes between time points or between races. Therefore, the *E. turcicum* response was investigated to identify which pathogenicity factors are activated by *E. turcicum* to propose mechanisms by which the fungus can cause disease. The *E. turcicum* effector complement was predicted using a bioinformatics approach and included known and putative effectors. Expression was confirmed for the known effector Ecp6 and the putative effector SIX13-like, and showed a trend of increased expression during biotrophy. Allelic variation in SIX13-like showed a host-specific pattern, while SIX5-like was conserved among isolates from different locations and hosts. In previous research, *formae speciales* of *F. oxysporum* could be distinguished based on polymorphisms among some of the SIX genes. We made a similar observation that host-specific patterns of the *SIX13-like* gene sequences support the host specialization of *E. turcicum* reported previously (Bergquist and Masias, 1974; Tang et al., 2015). Our study also revealed putative pathogenicity mechanisms employed by *E. turcicum* to cause disease and was the first report of the putative *E. turcicum* effector repertoire.

Disease development was similar to symptoms observed during previous studies of the *E. turcicum* infection process (Hilu and Hooker, 1964, 1965). The first disease symptoms were observed as light, chlorotic flecks at 2 dpi, which became more numerous at 5 dpi. Lesions became visible at 7 dpi and by 13 dpi; mature, sporulating lesions were visible. In previous microscopic investigations, initial symptoms appeared as small, light green to whitish flecks (Hilu and Hooker, 1964, 1965). Within the flecks, hyphae grew slowly toward the neighboring mesophyll cells and others grew toward the xylem (Hilu and Hooker, 1964). Small lesions spanning two to three veins were observed at 6 dpi, and expansion of chlorotic flecks were ascribed to slowly advancing hyphae in the chlorenchyma tissue. Lesions spanned 6–8 veins at 8 dpi and microscopic investigation revealed that growth of hyphae from the xylem into healthy neighboring tissue was responsible for lesion expansion (Hilu and Hooker, 1964). Although *E. turcicum* has been described as a hemibiotroph (Xue et al., 2013; Hurni et al., 2015), it is not known when the fungus switches from a biotrophic to a necrotrophic lifestyle. Based on the symptoms observed in this study as well as reports from previous studies, we hypothesize that during the infection of susceptible maize seedlings, *E. turcicum* switches from a biotrophic to a necrotrophic lifestyle between 5 and 8 dpi. In addition, the increase in fungal biomass from 5 to 7 dpi and peak expression of CWDEs at 13 dpi further support that, during this study, *E. turcicum* followed a biotrophic lifestyle for the first 7 days where after the onset of necrotrophy occurred.

Despite *E. turcicum* being described as a hemibiotroph, evidence to support the lifestyle strategy of this pathogen was lacking. Therefore, we further queried the *in planta* RNAseq data to determine if *E. turcicum* uses its own or host resources during early stages of infection. Investigation of the β-oxidation pathway indicated possible lack of expression for two enzymes, which suggests that *E. turcicum* is using host resources for growth during early stages of infection, which corresponds to a biotrophic infection strategy. In the *Zymospetoria tritici*, wheat interaction, genes encoding key enzymes required for the β-oxidation pathway were upregulated and indicated that the fungus is using internal fatty acid and lipid stores for energy generation during early stages of infection (Rudd et al., 2015). The authors hypothesized that *Z. tritici* follows a modified hemibiotrophic strategy, by suppressing host defenses at early time points before switching to a necrotrophic life stage (Rudd et al., 2015). Although our RNAseq data provide some evidence for a biotrophic phase in the lifestyle of *E. turcicum*, this conclusion is limited due to pooling of biological replicates. Further testing is required by sequencing the transcriptomes of biological replicates at these disease stages and can be supplemented by examining the host responses that occur at the same disease stages. For the purpose of further analyses and discussion, we assume *E. turcicum* is a hemibiotrophic pathogen.

In this study, peptidases with a putative function in pathogenicity were identified. Of particular interest are proteins similar to a leupeptin-inactivating enzyme and fungalysin. Leupeptin is a cysteine and serine protease inhibitor initially identified from *Actinomycetes* (Hozumi et al., 1972). Leupeptin-inactivating enzymes are produced by *Streptomyces exfoliates*, and a similar protein was identified from the tomato pathogen, *S. lycopersici* (Kim et al., 1998). Although a role for leupeptin-inactivating enzymes have not yet been established in fungi, the high expression levels of this gene warrant further investigation into the protein's function. In the maize pathogen *Fusarium verticillioides*, fungalysin was found to cleave within a sequence conserved in class IV chitinases (Naumann et al., 2011). In *Ustaligao maydis*, a fungalysin with a dual function in modulating both plant and fungal chitinases was identified (Ökmen et al., 2018). Mutants lacking fungalysin showed reduced virulence as well as impaired separation of haploid sporidia. Further investigation of the candidate identified in *E. turcicum* may reveal a role in modulating host responses through cleavage of maize chitinases. Functional characterization of the peptidases identified in this study may reveal the various mechanisms whereby *E. turcicum* is able to infect and colonize maize plants.

In this study, 36 of the 46 *in planta* expressed CWDEs were significantly expressed (read count values >2) only at 13 dpi, indicating that these may contribute to the induction of necrosis in maize. Transcriptional profiles of the CWDEs secreted by *Z. tritici* at distinct disease stages showed that CWDE expression was low during the asymptomatic phase, with the global peak expression detected during necrotrophy (Palma-Guerrero et al., 2017). This expression pattern was hypothesized to be due to the induction of necrotrophy by CWDEs through plant cell wall degradation and subsequent nutrient release needed for fungal metabolism.

Hemibiotrophic and biotrophic pathogens use toxins to gain access to nutrients after host colonization. Genes potentially involved in secondary metabolite biosynthesis identified during this study included StNPS6 and StNPS2, tentoxin synthase (*TES*), *HC-toxin* synthase 1 (*HTS1*), and homologs of the TOX genes. StNPS6 and StNPS2 are of interest as knockout mutants are reduced in virulence (Lee et al., 2005; Condon et al., 2013). NPS6 of *C. heterostrophus* was shown to be required for full virulence on maize (Lee et al., 2005), and NPS2 is highly conserved within the *Dothideomycetes* (Condon et al., 2013). Although *E. turcicum* has been shown to produce monocerin, genes of the biosynthetic cluster have not yet been elucidated (Cuq et al., 1993). This study showed that the *E. turcicum* expresses genes for proteins similar to characterized toxin synthases including the tentoxin synthase gene *TES* from *A. alternata* as well as the HC-toxin synthase gene, *HTS1*, from *C. carbonum*. *Cochliobolus miyabeanus* was the first non-*Alternaria* species shown to produce tentoxin (de Bruyne et al., 2016). Tentoxin occurs in many *Alternaria* species and is known to induce chlorosis due to damage to the F_1_-ATPase of chloroplasts in sensitive plants (Li et al., 2016). Although the *TES* gene is present in the *E. turcicum* genome, read count values were low and do not indicate that tentoxin plays a role in pathogenicity of *E. turcicum* on maize. Similarly, expression of *HTS1* was low or absent. In conjunction with *TOXA, TOXC, TOXD, TOXE, TOXF*, and *TOXG, HTS1* is involved in HC-toxin biosynthesis (Walton, 2006). Homologs of the *TOX* genes have been identified in *E. turcicum* (Ohm et al., 2012) although the lack of duplicates and organization of the genes on separate scaffolds suggest that the HC-toxin is not produced by this pathogen. However, read count values obtained in this study revealed expression of all *TOX* homologs and suggests that the HC-toxin may play a role in the pathogenicity of *E. turcicum* on maize. Identification and expression profiling of genes involved in monocerin biosynthesis as well as bioassays to identify toxins produced by *E. turcicum* will contribute to our understanding of the necrotrophic life stage of this pathogen.

The putative effector repertoire of *E. turcicum* was identified based on known effector characteristics. A total of 351 candidates were predicted, of which 346 showed evidence of expression from transcriptome sequencing. Proteins with significant similarity to known effectors, such as Ecp6, cerato-platanin, SIX13, and SIX5 were detected *in planta*. Expression profiling of *Ecp6* and *SIX13-like* showed a general trend of increased expression at 5 and 7 dpi as compared to the 2 and 13 dpi datasets. Sanz-Martín et al. (2016) also noted that *Ecp6* is highly expressed during the biotrophic phase of *Colletotrichum graminicola* infection of maize and low during necrotrophy (Sanz-Martín et al., 2016). The *SIX13-like* candidate effector is an interesting target for future studies to determine the effect(s) of this candidate on pathogenicity.

Interest in the candidate effectors *SIX13-like* and *SIX5-like* were further fueled by the presence of *E. turcicum* in the xylem of maize plants (Hilu and Hooker, 1964; Kotze et al., 2019). These candidates were uniquely expressed during *in planta* infection. Although 14 *SIX* genes have been identified from *F. oxysporum* (Houterman et al., 2007; Schmidt et al., 2013), only two *SIX* effectors (*SIX13* and *SIX5*) showed similarity to *E. turcicum* proteins. The *SIX* genes distinguish the *formae speciales* of *F. oxysporum* based on the presence/absence or sequence variation, and thus, variable numbers of *SIX* genes are found in *F. oxysporum* genomes (Lievens et al., 2009). *SIX* genes have been identified in five non-*Fusarium species*. Two of these species are known to invade host xylem cells during infection (*E. turcicum* and *V. nonalfalfae*) similar to *F. oxysporum*, while the other three species (*Ustilaginoidea virens, C. orbiculare*, and *C. gloeosporioides*) do not colonize the xylem (Pandey et al., 2012; Li et al., 2013; Yadeta and Thomma, 2013; Marton et al., 2018; Kotze et al., 2019). Although some of the *SIX* effectors are required for pathogenicity, lead to host responses upon recognition, and can interact with *R-*genes, the biological function of the majority of these genes is unknown (Ma et al., 2015; Niu et al., 2016). Only the function for *SIX5* is known, and it interacts with the effector *Avr2* at plasmodesmata to enable movement of *Avr2* to neighboring cells (Cao et al., 2018). It is possible that the *SIX13-like* and *SIX5-like* candidate effectors identified in this study are indeed secreted in the xylem of maize plants during maize infection, but it is unclear whether these candidates have a similar function to the *SIX* genes from *F. oxysporum*. Future investigations will analyze maize xylem sap to identify whether the SIX13-like and SIX5-like candidate effector proteins are present and will be followed by functional assays to determine whether knocking out the protein alters invasion success.

Gene expression was compared between *E. turcicum* race 13N and a race 23N isolates to identify genes expressed in only one of the races. Comparisons were also made with an *in vitro* grown race 13N isolate to identify the number of genes expressed *in planta* only. Obtained read count values indicated a greater number of genes shared between *in planta* groups (of different races) as compared to the same race under different conditions (*in planta* vs. *in vitro*). Transcriptomic comparisons between races of *F. oxysorum* f. sp. *cubense* able to infect the banana cultivars Gros Michel (race 1) or Cavendish (race 4) revealed that the most distinct differences were observed in expression patterns between the two races rather than in the numbers of differentially expressed genes (Qin et al., 2017). Therefore, transcriptome sequencing of biological replicates to identify genes differentially expressed between races of *E. turcicum* may elucidate differences in infection mechanisms. In addition, sequencing of a race 23N *in vitro* grown isolate should be included to identify genes expressed *in vitro* only for this race.

Comparison of effector profiles across races indicated limited race-specific effectors, with the majority of effectors expressed at all-time points. Of particular interest was *AVRHt1*, the putative effector interacting with the maize resistance gene *Ht1* (Mideros et al., 2018). Transcriptome data revealed expression of *AVRHt1* in the 23N dataset only. Owing to the size of the protein (4,039 aa), it does not conform to typical effector characteristics and was not identified as a candidate effector by the pipeline employed in this study. Previously, a PKS (*ACE1*) was identified in *M. oryzae*, which produces a secondary metabolite that is recognized by the rice resistance (*Pi33)* gene product (Böhnert et al., 2004; Collemare et al., 2008). Interaction between the secondary metabolite dependent on *ACE1* and the *Pi33*-gene product leads to the initiation of host defense responses, and disruption of *ACE1* abolishes recognition of the pathogen by the host. Despite the size of *ACE1* (4,035 aa), the secondary metabolite produced is regarded as an effector (Böhnert et al., 2004). Interestingly, the 23N isolate sequenced in this study encoded the same nucleotide at the same position as reported for race 2 isolates by Mideros et al. (2018). Furthermore, expression of *AVRHt1* was not observed in the 13N *in vitro* or the 13N *in planta* datasets. This supports the findings by Mideros et al. (2018) that *AVRHt1* is not expressed by race 1 (and race 1-related) *E. turcicum* isolates and that the secondary metabolite dependent on *AVRHt1* is recognized by the maize resistance gene *Ht1*. The candidate effectors identified in this study that were unique to race 23N represent interesting targets for further studies to identify the effector interacting with *Ht2*. Sequence comparisons of race 1 and 2 effector candidates may reveal additional candidates interacting with *Ht2*.

Sequence analysis revealed that *SIX13-like* showed host specificity, and the pattern observed within *SIX13-like* supports the hypothesis by Nieuwoudt et al. (2018) that *E. turcicum* isolates from maize and sorghum are genetically distinct. Our data were striking in that the *SIX13-like* sequences grouped by host and remained distinct between maize and sorghum isolates at each of the sites in South Africa. The collection sites Delmas and Greytown are in different agro-ecological zones and 400 km apart, indicating that growing environment had minimal influence on selection of *SIX13-like* sequences compared to host species. The characteristics of the SIX13-like protein support our hypothesis that SIX13-like is an effector. In future, knockout studies can be conducted to determine the contribution of *SIX13-like* to the pathogenicity of *E. turcicum*. Whether the polymorphisms observed in *SIX13-like* enable evasion of host recognition or contribute to host specificity of *E. turcicum* can be elucidated by targeted mutation of host-specific amino acid sites.

Sequence variation was limited in *SIX5-like*, and similarly, sequencing of *Ecp6* across a diverse set of *E. turcicum* isolates from South Africa revealed no sequence variation (BG Crampton, personal communication). Sequence variation was rare and more often observed in non-protein coding regions of *Ecp1, Ecp2, Ecp4*, and *Ecp5* in a global set of *C. fulvum* isolates from Europe, North and South America, Japan, New Zealand, and Zimbabwe (Stergiopoulos et al., 2007). Although the cognate tomato *R-*genes interacting with the *C. fulvum* Ecp effectors have been identified, these have not been widely used in commercially grown tomatoes to manage leaf mold resulting from infection with the pathogen (Thomma et al., 2005; Stergiopoulos et al., 2007). Absence of these genes in tomato breeding programs is postulated to be the reason for the high conservation in the *Ecp* genes. Therefore, it is possible that the lack of sequence variation in *SIX5-like* is due to the absence of a corresponding *R*-gene in commercially grown maize fields in South Africa. Alternatively, the lack of sequence variation in *SIX5-like* may indicate that this candidate plays a crucial role in pathogenicity and that mutations are not well-tolerated. Further investigation is required to confirm which hypothesis is true.

Transcriptional profiles obtained for peptidases, cell wall degrading enzymes, and secondary metabolite enzymes of *E. turcicum* during infection of maize seedlings revealed interesting candidates for functional investigations. Furthermore, the effector complement of *E. turcicum* was identified and contains both known and putative novel effectors. The gene expression analysis conducted revealed evidence of effector expression at 5 and 7 dpi, corresponding to the biotrophic stage of *E. turcicum* infection of maize seedlings, as seen from fungal quantification and percentage of reads mapped. Fungal growth *in planta* was low at 2, 5, and 7 dpi, where after extensive colonization of host tissues occurred. In this study, the switch from biotrophy to necrotrophy of *E. turcicum* infection of maize seedlings occurred after 7 dpi based on fungal growth, and possibly at 8 dpi, but further studies are required to confirm this hypothesis. In future, closer inspection of this window may reveal with greater clarity the biotrophy–necrotrophy switch of *E. turcicum*. Furthermore, host-specific SNPs detected in *SIX13-like* indicates genetic isolation between isolates from different hosts. In the future, pathogenicity trials of genetically distinct *E. turcicum* isolates identified here will be conducted to investigate whether the observed genetic separation between isolates from maize and sorghum corresponds to host specificity.

## Data Availability Statement

The raw *in vitro* and *in planta* RNA-seq reads generated for this study can be found in the GenBank Short Read Archive under the accession number PRJNA560644. The sequences generated for *E. turcicum* sequences of SIX13-like and SIX5-like in this study are available in the GenBank database under the following accession numbers: MN219490–MN219509 and MN 334674–MN334695.

## Author Contributions

BC, MH, and DB contributed to the experimental design. MH conducted all experimental work and analyses. MH wrote the manuscript with critical review and inputs from BC and DB.

### Conflict of Interest

The authors declare that the research was conducted in the absence of any commercial or financial relationships that could be construed as a potential conflict of interest.
