# Supplementary material for: Time-Course RNAseq Reveals Exserohilum turcicum Effectors and Pathogenicity Determinants
Source: Front Microbiol. 2020 Mar 20;11:360. doi: 10.3389/fmicb.2020.00360 (PMC7099616; doi:10.3389/fmicb.2020.00360)
Supplement: Supplementary file 1 [file Table_1.DOCX]

Supplementary Table 1 Oligonucleotide primers used during this study.

| **Reason for primer** | **Primer identifier** | **Protein Identifier^c^** | **Sequence (5’ – 3’)** | **Amplicon size (bp)** |
| --- | --- | --- | --- | --- |
| Reference^a^ | 40S F | 168532 | TCCACGACGCCATTCTG | 100 |
|  | 40S R |  | AGAACGACCTTGAGGATCTTG |  |
| Reference^a^ | EF1-α F | 36922 | ACCGTTACCAGGAGATCATC | 109 |
|  | EF1-α R |  | ATGTTGTCGCCGTTGAAG |  |
| Reference^a^ | GAPDH F | 183995 | TCCACTCCTACACTGCTACC | 99 |
|  | GAPDH R |  | TGCTGCTGGGAATGATGTT |  |
| Target^a^ | SIX13-like F | 34559 | GTCGGTGTTGAACGGGAATA | 116 |
|  | SIX13-like R |  | TGAAGGTAAATGGTGCCTACG |  |
| Target^a^ | Ecp6 F | 136414 | GCAACGACACCTGTGTCAAC | 148 |
|  | Ecp6 R |  | AAGTTGGGGTTTGTGACCTG |  |
| Sequencing^b^ | SIX13-like_F | 34559 | CCAGACCCACAGATGTAGATAA | 1009 |
|  | SIX13-like_R |  | CATTGACCGAAACCTCTTCA |  |
| Sequencing^b^ | SIX5_F | 30080 | ATCTCTCTCTGCTTCCTTACTC | 752 |
|  | SIX5_R |  | AAGACGAAAGACGACCCTAT |  |

^a^ Used in RT-qPCR

^b^ Used for gDNA sequencing

^c^ *Exserohilum turcicum* protein identifier from <http://genome.jgi.doe.gov/Settu1/Settu1.home.html>.
