# Supplementary figures and images for: Time-Course RNAseq Reveals Exserohilum turcicum Effectors and Pathogenicity Determinants
Source: Front Microbiol. 2020 Mar 20;11:360. doi: 10.3389/fmicb.2020.00360 (PMC7099616; doi:10.3389/fmicb.2020.00360)

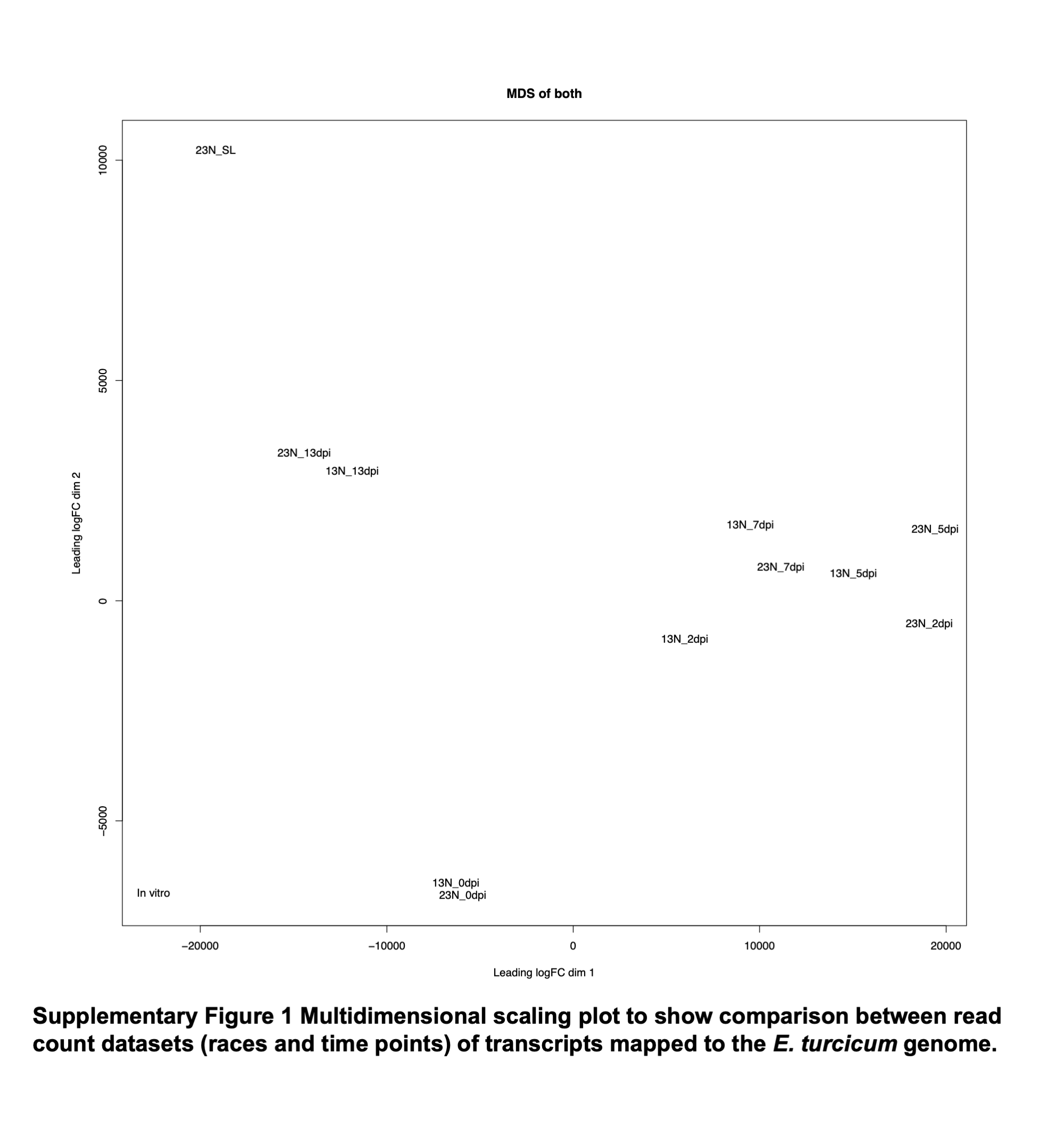

Supplement: Supplementary file 10 [file Image_1.TIFF]

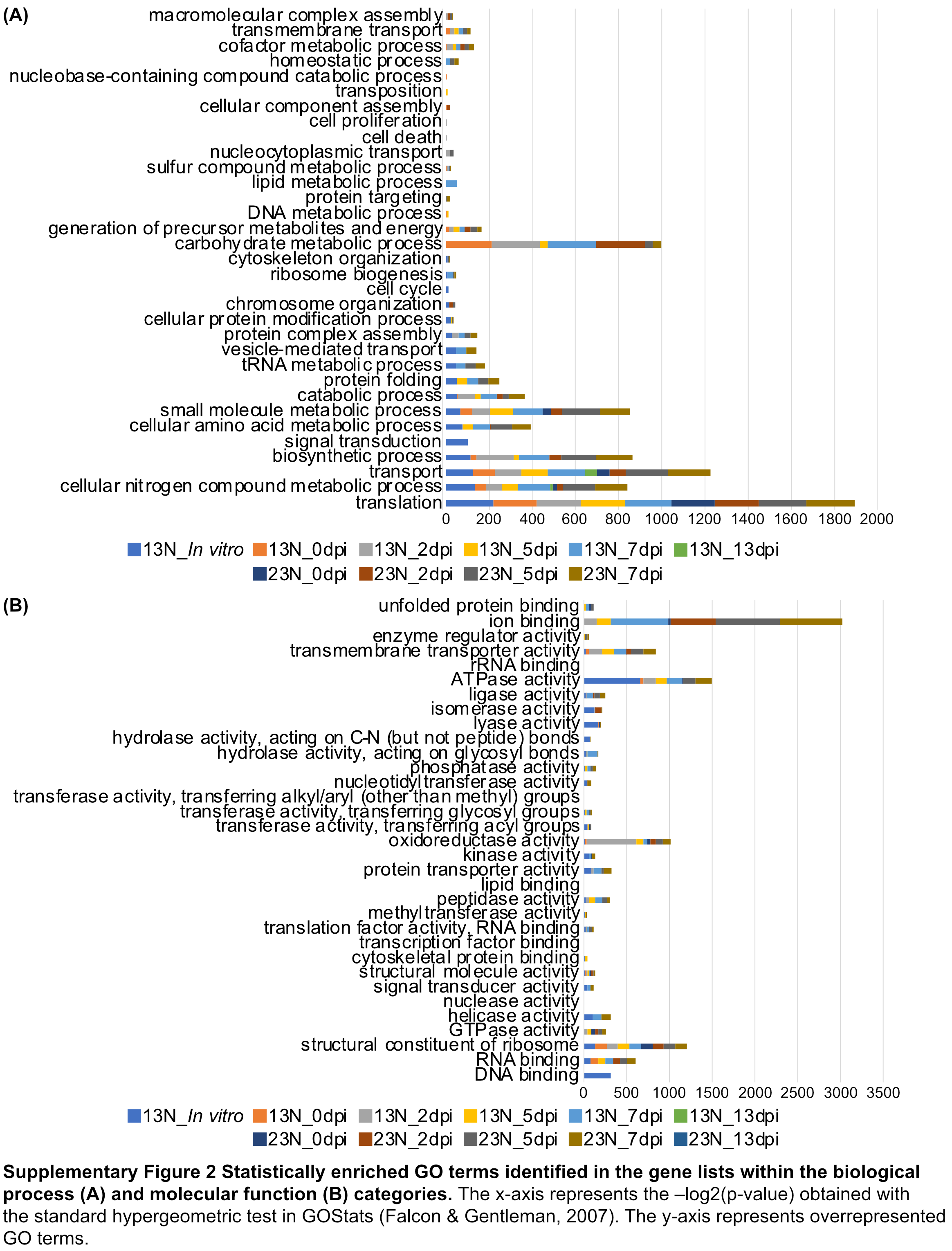

Supplement: Supplementary file 11 [file Image_2.TIFF]

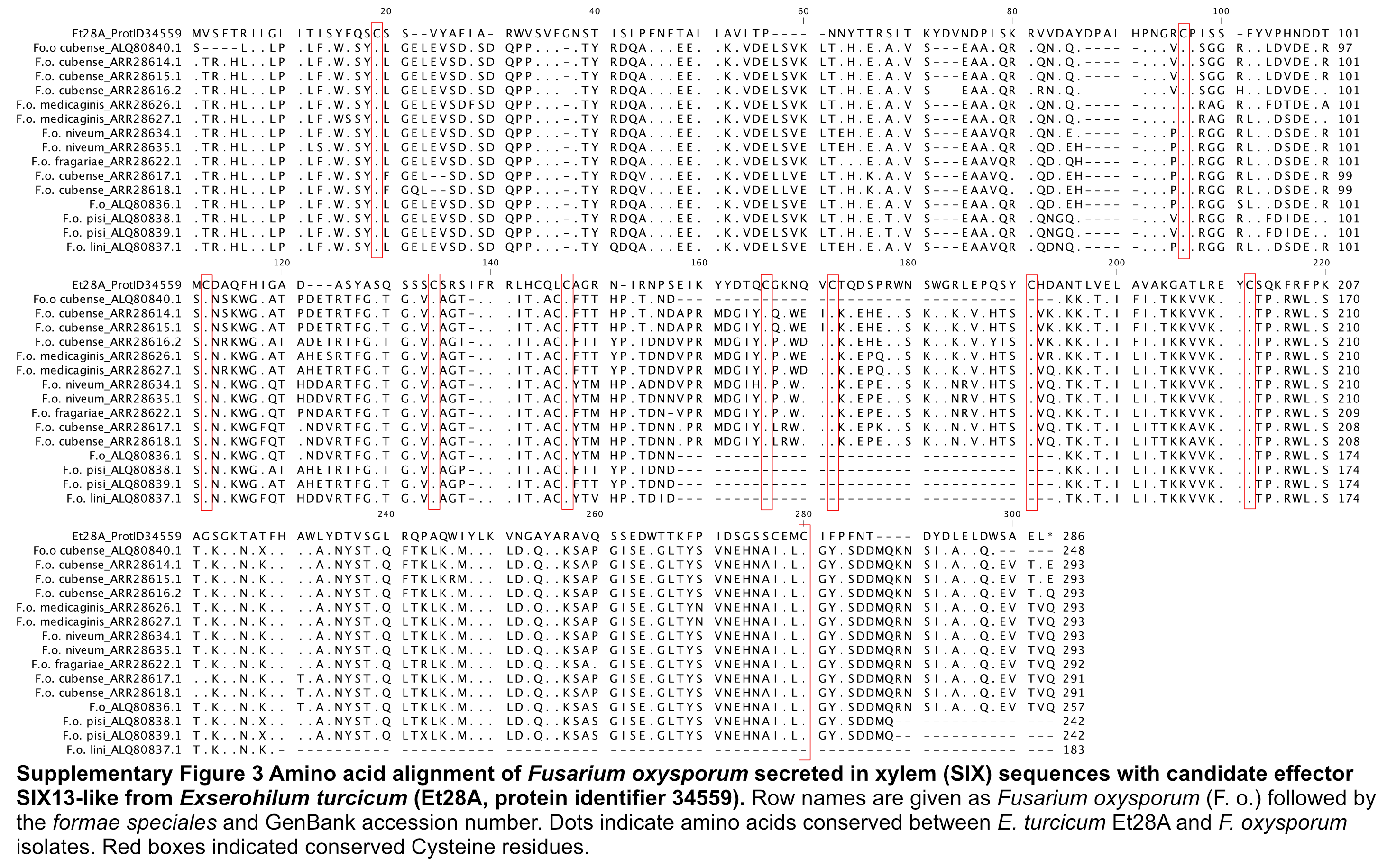

Supplement: Supplementary file 12 [file Image_3.TIFF]

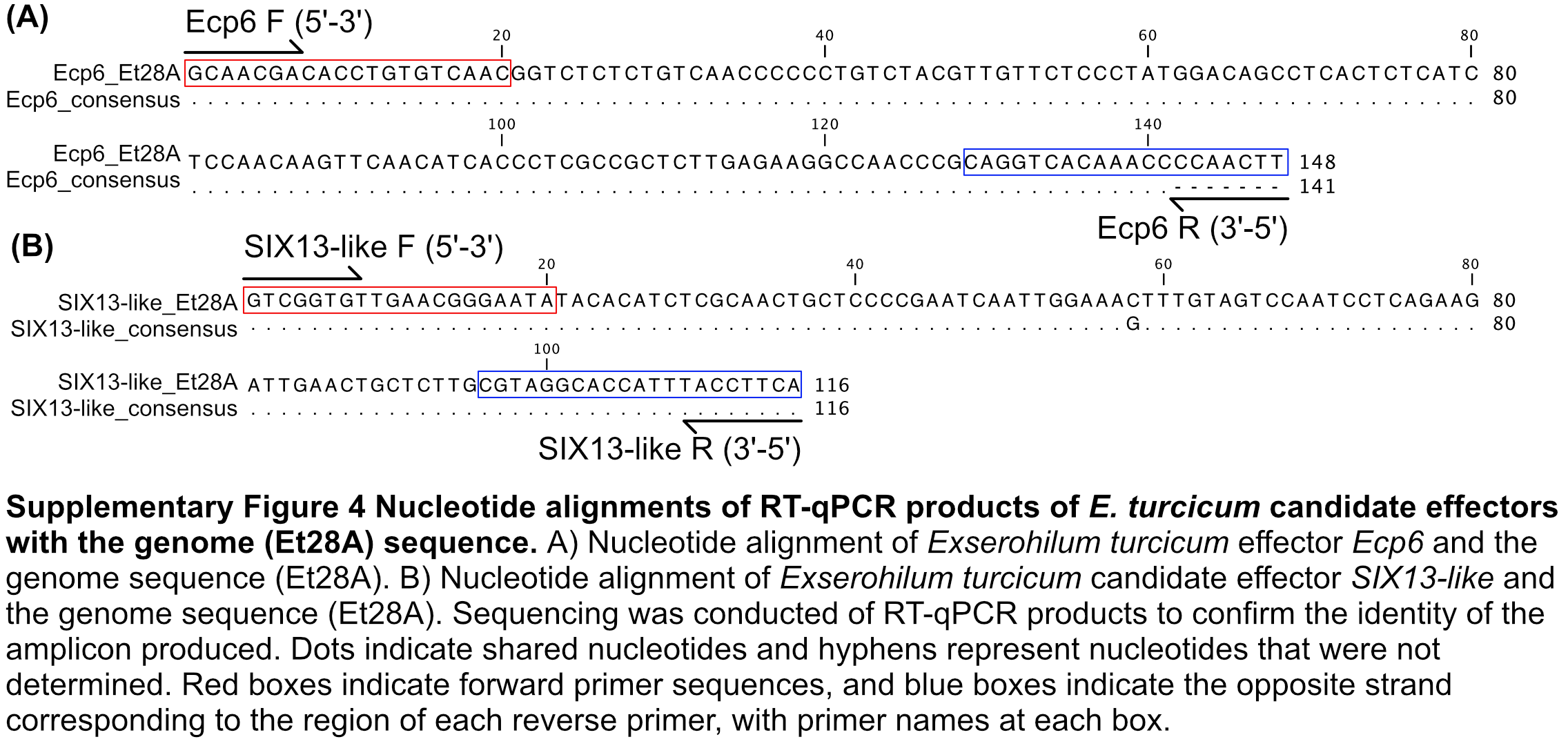

Supplement: Supplementary file 13 [file Image_4.TIFF]

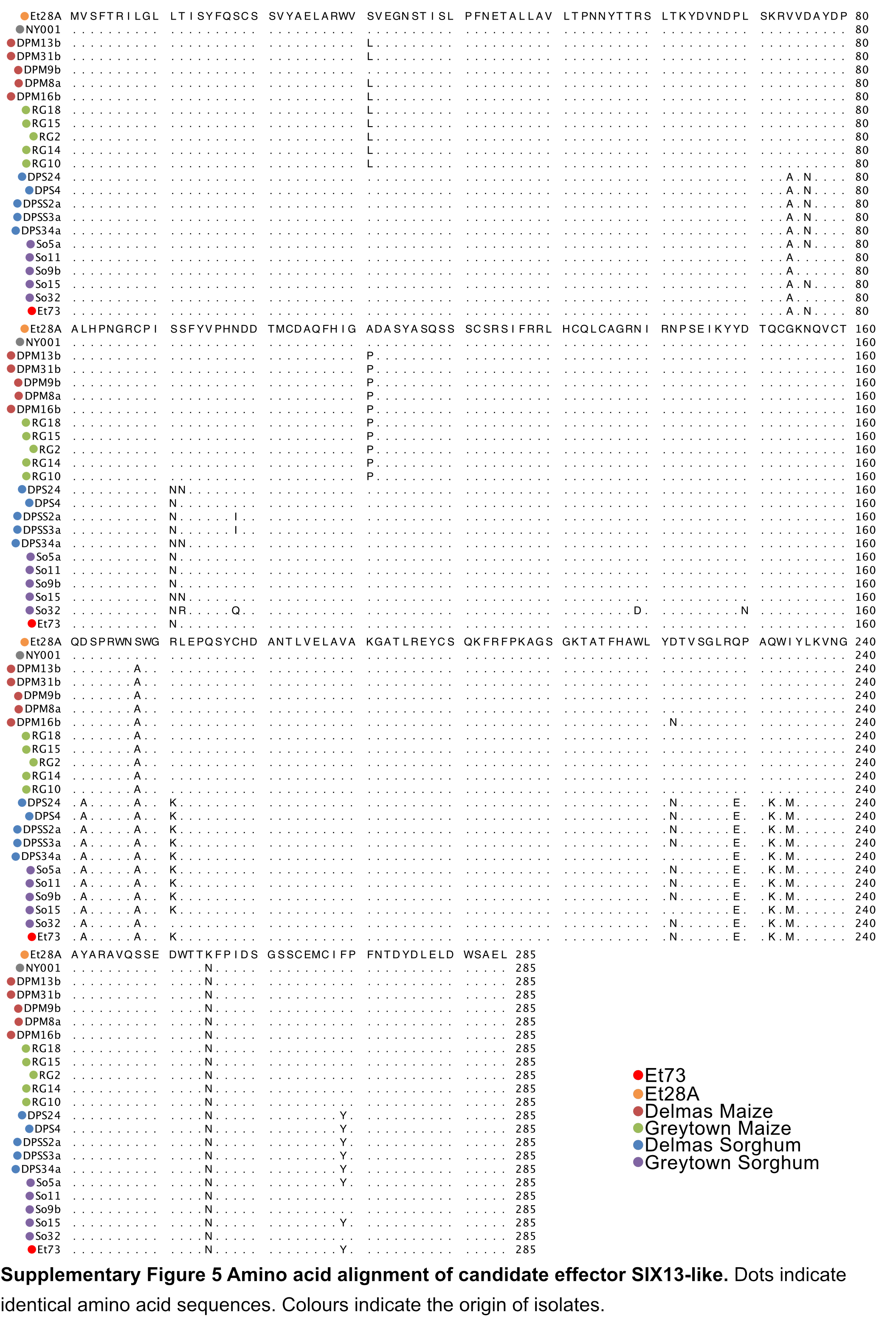

Supplement: Supplementary file 14 [file Image_5.tiff]
